# m^6^A‐Dependent Modulation via IGF2BP3/MCM5/Notch Axis Promotes Partial EMT and LUAD Metastasis

**DOI:** 10.1002/advs.202206744

**Published:** 2023-05-12

**Authors:** Xia Yang, Qiaorui Bai, Weizhong Chen, Jiaer Liang, Fang Wang, Weiqi Gu, Lei Liu, Quanfeng Li, Zishuo Chen, Anni Zhou, Jianting Long, Han Tian, Jueheng Wu, Xiaofan Ding, Ningning Zhou, Mengfeng Li, Yi Yang, Junchao Cai

**Affiliations:** ^1^ Advanced Medical Technology Center The First Affiliated Hospital Zhongshan School of Medicine Sun Yat‐Sen University Guangzhou 510080 China; ^2^ Department of Pharmacology Zhongshan School of Medicine Sun Yat‐Sen University Guangzhou 510080 China; ^3^ Chongqing Key Laboratory of Molecular Oncology and Epigenetics The First Affiliated Hospital of Chongqing Medical University Chongqing 400016 China; ^4^ Department of Orthopedics The Eighth Affiliated Hospital Sun Yat‐Sen University Guangzhou 518033 China; ^5^ Cancer Institute Southern Medical University Shenzhen 510515 China; ^6^ Department of Oncology The First Affiliated Hospital Sun Yat‐sen University Guangzhou 510080 China; ^7^ Faculty of Health Sciences Building University of Macau Macau 999078 China; ^8^ Department of Medical Oncology Sun Yat‐Sen University Cancer Center Guangzhou 510060 China; ^9^ Key Laboratory of Tropical Disease Control (Sun Yat‐sen University) Ministry of Education Guangzhou 510080 China

**Keywords:** lung adenocarcinoma, m^6^A modification, MCM5, Notch signaling, partial EMT

## Abstract

The importance of mRNA N6‐methyladenosine (m^6^A) modification during tumor metastasis is controversial as it plays distinct roles in different biological contexts. Moreover, how cancer cell plasticity is shaped by m^6^A modification is interesting but remains uncharacterized. Here, this work shows that m^6^A reader insulin like growth factor 2 mRNA binding protein 3 (IGF2BP3) is remarkably upregulated in metastatic lung adenocarcinoma (LUAD) and indicates worse prognosis of patients. Interestingly, IGF2BP3 induces partial epithelial‐mesenchymal‐transition (EMT) and confers LUAD cells plasticity to metastasize through m^6^A‐dependent overactivation of Notch signaling. Mechanistically, IGF2BP3 recognized m^6^A‐modified minichromosome maintenance complex component (MCM5) mRNAs to prolong stability of them, subsequently upregulating MCM5 protein, which competitively inhibits SIRT1‐mediated deacetylation of Notch1 intracellular domain (NICD1), stabilizes NICD1 protein and contributes to m^6^A‐dependent IGF2BP3‐mediated cellular plasticity. Notably, a tight correlation of the IGF2BP3/MCM5/Notch axis is evidenced in clinical LUAD specimens. Therefore, this study elucidates a critical role of m^6^A modification on LUAD cell plasticity in fostering tumor metastasis via the above axis, providing potential targets for metastatic LUAD.

## Graphical abstract

IGF2BP3, remarkably upregulated in metastatic lung adenocarcinoma (LUAD), induces partial‐EMT, and confers LUAD cells plasticity to metastasize through m^6^A‐dependent manner. IGF2BP3 recognizes and stabilizes m^6^A‐modified MCM5 mRNAs, leading to enhanced Notch signaling activity and cellular plasticity. This study elucidates a critical role of m^6^A modification in shaping cancer cell plasticity via the IGF2BP3/MCM5/Notch axis.

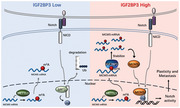

## 1 Introduction

Lung cancer remains the most frequently diagnosed and one of the deadliest cancer types worldwide. Metastasis can be found locally or distantly in nearly two‐thirds of lung cancer patients at the time of initial diagnosis. As a major histopathological subtype, lung adenocarcinoma (LUAD) presents the highest risk of metastasis incidence and can metastasize rapidly to lymph nodes, contralateral lung and multiple distant organs. Despite significant advancements made in therapeutic effectiveness against LUAD in recent decades, the long‐term prognosis for metastatic LUAD patients remains challenged with a median survival time of merely 5 months and over a half of metastatic patients die within 1 year.^[^ ^1^, ^2^ ^]^ Metastatic LUAD patients mostly display poor responsiveness to currently available treatments and high frequencies of post‐treatment relapse. It is therefore important to identify new, effective therapeutic targets for metastatic LUAD.

Metastasis accounts for more than 90% of cancer‐related deaths, and acquisition of metastatic properties is essential for tumor cell dissemination. During the multi‐step process of metastasis, reversible phenotype transitions of cell states are usually required. The ability of cells to undergo molecular and phenotypic changes is a phenomenon known as cellular plasticity, and this pliability in cell states helps tumor cells to cope with selective pressures and facilitates distant metastasis.^[^ ^3^ ^]^ Epithelial‐to‐mesenchymal transition (EMT), a process by which epithelial cells lose cell polarity and cell‐cell adhesion and gain fibroblast‐like mesenchymal properties, is such an important cellular plasticity that empowers tumor cells potent invasive and metastatic traits.^[^ ^4^, ^5^ ^]^ However, it appears that evidence of complete conversion from epithelial to mesenchymal state is scarce in either primary or secondary tumor tissues. Interestingly, it is suggested that an intermediate state, namely, partial EMT (p‐EMT), at which tumor cells behave with the mesenchymal state but retain epithelial features, endows tumor cells with a greater potential for both dissemination and colonization.^[^ ^5^, ^6^ ^]^ However, the precise contextual signals giving rise to p‐EMT state requires further investigation. Therefore, elucidating how cancer cell plasticity is regulated to promote metastasis should be helpful for further understanding the metastasis process and developing therapeutic approaches.

Both genetic and epigenetic alterations importantly regulate tumor cell plasticity and metastasis.^[^ ^6^, ^7^ ^]^ Recently, global detection of RNA modifications reveals the importance of N^6^‐methyladenosine (m^6^A) modification, the most abundant mRNA modification manner, in a variety of physiological and pathological processes. The m^6^A modification is dynamically regulated by a “writer” complex (methyltransferase) normally consisting of METTL3, METTL14, and WTAP and “eraser” (RNA demethylase) proteins such as ALKBH5 and FTO. The m^6^A modified sites on RNA transcripts are recognized by “reader” proteins such as YTHDs and IGF2BPs, which can determine the cellular fate of target mRNAs through regulating splicing, nuclear exportation, degradation, stabilization, and translation of RNA molecules.^[^ ^8^, ^9^ ^]^ Notably, the importance of m^6^A during tumor metastasis is controversial as m^6^A modulators, including writers, erasers and readers, can cause opposite outcomes in different genetic and cellular contexts.^[^ ^10^, ^11^, ^12^, ^13^ ^]^ Therefore, it is necessary to clarify which of these m^6^A regulators can be both clinically and biologically important in promoting metastasis and how RNA m^6^A modification might shape cell plasticity during tumor metastasis.

The Notch signaling pathway is commonly overactivated during tumor progression.^[^ ^14^, ^15^ ^]^ Upon activation by ligands, Notch proteins can be cleaved to release its intracellular domain (NICD) into the nucleus, where it transactivates downstream target genes such as HES1 and HEY1. Accumulation of NICD proteins represents a hallmark of Notch signaling overactivation. However, under physiological conditions, NICD proteins are usually too short‐lived to achieve optimal signal intensity, and their half‐lives are fine‐tuned via ubiquitin‐proteasomal degradation regulated by post‐translational modifications, such as phosphorylation and acetylation.^[^ ^16^, ^17^ ^]^ Genomic alterations for Notch hyperactivation are frequently observed in patients with leukemia, colorectal, gastric, pancreatic and endometrial cancers, but are rare in either metastatic or primary LUAD.^[^ ^18^, ^19^ ^]^ Notably, direct m^6^A RNA modifications on Notch signaling components are controversial as their increased m^6^A levels can both promote and repress Notch signaling.^[^ ^20^, ^21^ ^]^ Therefore, how m^6^A modulation mediates Notch deregulation, especially in metastatic LUAD remains to be clarified.

In this study, we demonstrate that a critical m^6^A reader, insulin like growth Factor 2 mRNA binding protein 3 (IGF2BP3), is able to induce p‐EMT and empowers LUAD cells with high plasticity to metastasize through m^6^A‐dependent overactivation of Notch signaling. IGF2BP3 directly recognizes m^6^A modified mRNAs of minichromosome maintenance complex component 5 (MCM5) gene, a member of an evolutionarily conserved MCM family assisting in loading DNA onto replication origins,^[^ ^22^, ^23^ ^]^ to stabilize MCM5 mRNAs and to increase MCM5 protein expression, which binds NICD1, competitively abrogates SIRT1‐mediated NICD1 deacetylation and degradation, and potently induces cancer cell plasticity to facilitate metastasis in an m^6^A‐dependent manner. Therefore, our current study provides insights into m^6^A modulation on cancer cell plasticity and Notch1 signaling overactivation and potential therapeutic targets for metastatic LUAD.

## 2 Results

### 2.1 IGF2BP3 Induces p‐EMT and Promotes LUAD Metastasis via m^6^A Modification

**Figure 1:**
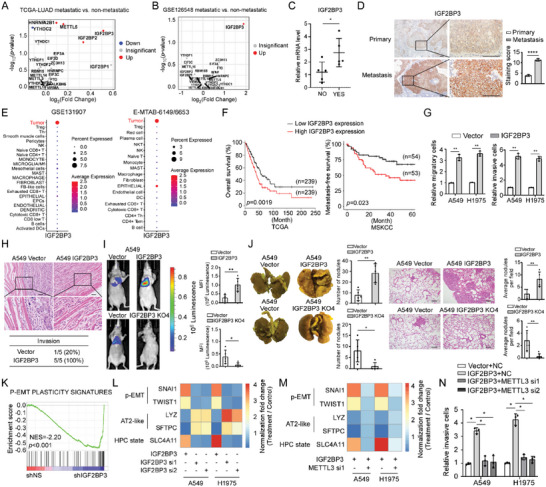
IGF2BP3 induces p‐EMT‐related cancer cell plasticity to facilitate metastasis depending on m6A modification. A,B) Expression of m^6^A‐related genes in indicated datasets. C) IGF2BP3 expression in primary tumors from 5 non‐metastatic (NO) and 5 metastatic (YES) LUAD patients. D) Representative images of IHC staining in primary and matched brain metastases. *n* = 10. Scale bar: 50 µm. E) The expression pattern in various cell types of IGF2BP3 is visualized by the Seurat *DotPlot* function. F) Overall and metastasis‐free survival based on the TCGA LUAD and MSKCC datasets, respectively. G) Transwell assays showing metastasis‐related traits. H) H&E staining shows muscle penetration in subcutaneous tumor xenografts formed by indicated cells (1 × 10^6^). The number of mice with local invasion was counted. *n* = 5, Scale bar: 50 µm. I,J) Bioluminescent images, picric acid staining and H&E staining show lung metastases formed by tail vein injection with indicated cells. The bioluminescent intensities and the number of lung surface nodules and metastatic lesions in mouse lung tissue were provided. *n* = 5, Scale bar: 50 µm. (K) GSEA of GSE90684 datasets. L,M) qRT‐PCR analysis of markers related to p‐EMT and lineage plasticity. N) Transwell assays showing the effects of silencing METTL3 on IGF2BP3‐induced pro‐metastatic traits. Statistical analyses were performed using a two‐tailed Student's *t*‐test, *: *p* < 0.05, **: *p* < 0.01, ****: *p* < 0.0001.

Through analyzing expression of 24 well‐known m^6^A writers, erasers and readers in metastatic LUAD tissue, we found that the TCGA LUAD datasets exhibited significant alterations of 4 readers (YTHDC2, HNRNPA2B1, IGF2BP2, and IGF2BP3) in metastatic versus non‐metastatic tumors (**Figure** **1**A). In the LUAD dataset GSE126548, only IGF2BP3 is significantly up‐regulated in metastatic tumors (Figure 1B). The upregulation of IGF2BP3 was validated in not only primary tumors derived from metastatic LUAD patients, but also in metastatic lesions as compared to their paired primary lung tumors as collected by this study (Figure 1C,D). Interestingly, via analyzing single‐cell RNA (scRNA) sequencing data,^[^ ^24^, ^25^ ^]^ we found that expression levels of IGF2BP3 in LUAD tumor cells were dramatically higher than those in any other cell type, indicating unique high‐level IGF2BP3 specifically expressed in LUAD tumor cells (Figure 1E), and that IGF2BP3 closely correlated with cancer metastasis signature at both single‐cell level and bulk‐tumor level via exploring scRNA‐seq‐based CancerSEA dataset and TCGA LUAD datasets, respectively (Figure S1A,B, Supporting Information). Importantly, high‐level IGF2BP3 indicated shorter overall and metastasis‐free survival (Figure 1F), suggesting an association of IGF2BP3 expression with LUAD metastasis.

Indeed, overexpressing IGF2BP3 strikingly promoted, whereas silencing IGF2BP3 abrogated migration and invasion of LUAD cells (Figure 1G and Figure S1C–E, Supporting Information). Subcutaneous tumor xenografts formed by IGF2BP3‐overexpressing A549 cells displayed penetration of tumor cells into neighboring subcutaneous tissue, which was barely observed in the similarly sized tumor xenografts formed by vector‐control A549 cells (Figure 1H). Intravenous injection of IGF2BP3‐overexpressing A549 cells generated many more cancerous lesions in various lobes of the lungs and depletion of IGF2BP3 in A549 cells almost totally abrogated lung metastasis (Figure 1I,J). Similarly, when injected intracardiacally, IGF2BP3‐overexpressing A549 cells developed systemic metastases in various organs including the lungs, limb bones, and brain, with metastasis incidence ranging from 20% to 60%, whereas vector‐control A549 cells basically metastasized in the limb bones of only 20% nude mice (Figure S1F,G, Supporting Information). Despite the potent pro‐metastatic role, IGF2BP3 did not induce EMT morphology in LUAD cells. Interestingly, IGF2BP3 overexpression significantly induced, whereas silencing IGF2BP3 inhibited expression of partial EMT (p‐EMT) signature and p‐EMT related transcription factors (Figure 1K,L).^[^ ^26^, ^27^, ^28^ ^]^ Meanwhile, lineage plasticity, which is thought to be important for evolution of metastatic subclones, was also regulated by IGF2BP3 (Figure 1L), as evidenced by IGF2BP3 overexpression‐induced loss of, and IGF2BP3 knockdown‐caused acquisition of alveolar‐type2‐like (AT2‐like) state.^[^ ^29^ ^]^ Expression of SLC4A11, a specific marker for high‐plasticity cell state in LUAD,^[^ ^30^ ^]^ was increased by IGF2BP3 overexpression but reduced by IGF2BP3 depletion (Figure 1L). Notably, silencing the crucial m^6^A writer METTL3 markedly attenuated IGF2BP3‐induced expression of p‐EMT and cell plasticity signatures (Figure 1M). In consistence, silencing METTL3 largely compromised the pro‐metastatic effects of IGF2BP3; unlike wild‐type METTL3, mutant METTL3 deprived of m^6^A catalytic activity failed to augment the pro‐metastatic effects of IGF2BP3 (Figure 1N and Figure S1H, Supporting Information). Taken together, these data suggest that IGF2BP3 induces p‐EMT to potently confer LUAD cells plasticity to metastasize in an m^6^A ‐dependent manner.

### 2.2 IGF2BP3 Fosters Cancer Cell Plasticity to Metastasize by Overactivating Notch1 Signaling

**Figure 2:**
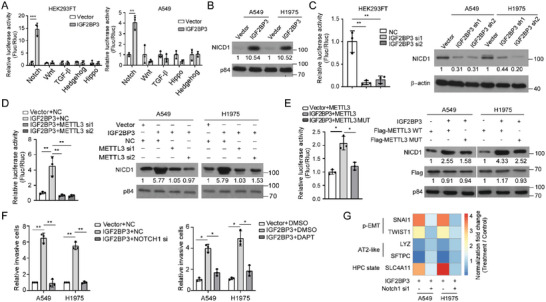
IGF2BP3 fosters cancer cell plasticity to metastasize by overactivating Notch1 signaling. A) Luciferase reporter assays show the effects of IGF2BP3 on metastasis‐related signaling pathways. B,C) WB and luciferase reporter assays examining Notch activity and NICD1 levels. D,E) Luciferase reporter and WB assays examining the effect of silencing or mutating METTL3 on Notch activation. F) Transwell assays show reversing effects of silencing or inhibiting Notch1 on IGF2BP3‐induced pro‐metastatic traits. G) qRT‐PCR analysis of markers related to p‐EMT and lineage plasticity. Error bars represent the means ± SD derived from three independent experiments. Statistical analyses were performed using a two‐tailed Student's *t*‐test, *: *p* < 0.05, **: *p* < 0.01, ***: *p* < 0.001.

Among several metastasis‐related signaling pathways, Notch signaling was overactivated by ectopic IGF2BP3 expression (**Figure** **2**A and Figure S2A, Supporting Information). IGF2BP3 markedly enhanced expression of NICD1 (Figure 2B), an active form of NOTCH1 protein cleaved by *γ*‐secretase when canonical Notch signaling is initiated. By contrast, IGF2BP3 depletion in LUAD cells remarkably inhibited Notch transactivation, decreased NICD1 levels and repressed expression of Notch downstream genes (Figure 2C, Figure S2B,C, Supporting Information) Notably, IGF2BP3‐induced Notch signaling activation could be dramatically impaired by silencing writers essential for m^6^A modification on mRNAs (such as METTL3, METTL14, and WTAP),^[^ ^8^, ^9^, ^31^ ^]^ but not altered by silencing METTL16, which mostly installs m^6^A onto the U6 small nuclear RNA (Figure 2D, Figure S2D,E, Supporting Information).^[^ ^9^, ^31^ ^]^ In addition, unlike wild‐type METTL3, mutant METTL3 loss of m^6^A catalytic activity failed to enhance IGF2BP3‐induced activation of Notch signaling in LUAD cells (Figure 2E and Figure S2F, Supporting Information). Furthermore, abrogating Notch signaling by silencing NOTCH1 or *γ*‐secretase inhibitor DAPT mitigated IGF2BP3‐promoted invasion and migration (Figure 2F and Figure S2G, Supporting Information). Moreover, inhibiting the Notch signaling via NOTCH1 knockdown reversed IGF2BP3‐induced expression of genes related to p‐EMT and cancer cell plasticity (Figure 2G). These data suggest that IGF2BP3 overactivates Notch1 signaling in an m^6^A‐dependent manner to promote cancer cell plasticity to metastasize.

### 2.3 IGF2BP3 Reads m^6^A‐Modified MCM5 mRNA to Promote its Stability

**Figure 3:**
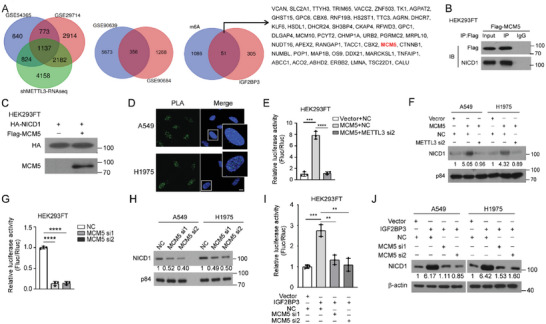
MCM5 contributed to IGF2BP3 activated Notch signaling in m^6^A dependent manner. A) Overlapping the RNA‐seq dataset of METTL3‐silenced A549 cells with two distinct MeRIP‐seq datasets (GSE29714 and GSE54365) reveals 1137 transcripts (left) and overlapping IGF2BP3‐RIP‐seq (GSE90639) and shIGF2BP3‐RNA‐seq (GSE90684) datasets reveals 356 transcripts (middle). 51 genes are IGF2BP3‐recognized and m^6^A‐modified transcripts, among which only MCM5 emerges as a potential NICD1‐interactive partner (right). B) Co‐IP assay shows interaction between MCM5 and NICD1 proteins. C) Pull‐down assay demonstrates a direct binding between recombinant MCM5 and NICD1. D) Representative images show the interaction and subcellular co‐localization of MCM5 and NICD1 proteins in LUAD cells. Scale bar: 5 µm. E–J) Luciferase reporter and WB assays examining Notch activity and NICD1 levels. All experiments were repeated three times with similar results. Statistical analyses were performed using a two‐tailed Student's *t*‐test, **: *p* < 0.01, ***: *p* < 0.001, ****: *p* < 0.0001.

We then elucidated how IGF2BP3 m^6^A‐dependently overactivates the Notch signaling. Among 8301 genes whose expression was significantly altered by METTL3 knockdown in A549 cells, 1137 gene transcripts were implicated to possess direct m^6^A modification by overlapping the RNA‐seq dataset of METTL3‐silenced A549 cells with two distinct MeRIP‐seq datasets (GSE29714 and GSE54365) (**Figure** **3**A, left panel). Meanwhile, overlapping public IGF2BP3‐RIP‐seq (GSE90639) and shIGF2BP3‐RNA‐seq (GSE90684) datasets revealed 356 transcripts recognized and regulated by IGF2BP3 (Figure 3A, middle panel), among which 51 genes were shared by the 1137 m^6^A ‐modified transcripts (Figure 3A, right panel). Since IGF2BP3 increased NICD1 levels and overactivated Notch signaling, by further analyzing published data of tandem affinity chromatography and mass spectrometry of NICD1‐interacting proteins,^[^ ^32^ ^]^ we identified MCM5 as the only potential IGF2BP3‐recognized target gene that also interacts with NICD1. Indeed, interactive binding assays showed direct interaction between MCM5 and endogenous NICD1 and nuclear co‐localization in LUAD cells (Figure 3B–D and Figure S3A, Supporting Information). Overexpressing MCM5 in LUAD cells indeed overactivated Notch signaling and increased NICD1 levels in a fashion dependent on m^6^A modification (Figure 3E,F and Figure S3B, Supporting Information), and knockdown or knockout of MCM5 displayed opposite effects (Figure 3G,H and Figure S3C–F, Supporting Information). Furthermore, silencing MCM5 diminished IGF2BP3‐activated Notch signaling (Figure 3I,J and Figure S3G, Supporting Information).

**Figure 4:**
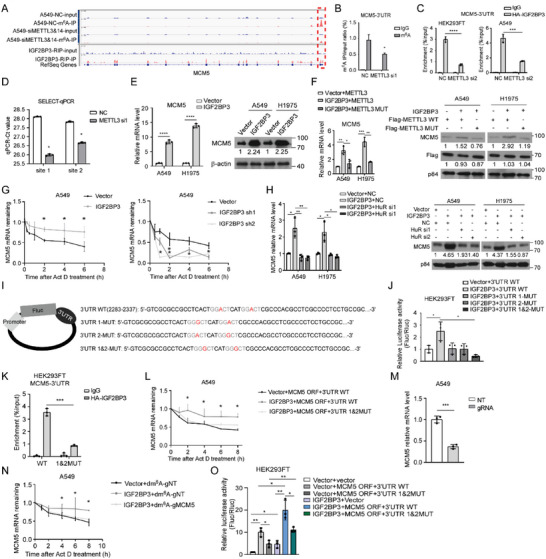
IGF2BP3 reads m6A‐modified MCM5 mRNA to promote its stability. A) Analysis of m^6^A modification peak based on IGF2BP3‐RIP‐seq (GSE90639) and MeRIP‐seq (GSE54365) datasets. B) MeRIP‐qPCR assay demonstrates enrichment of m^6^A modification in MCM5 3′UTR in A549 cells silenced with control (NC) or METTL3. C) RIP‐qPCR detecting the binding of IGF2BP3 with MCM5 mRNA 3′UTR in A549 and HEK293FT cells. D) The threshold cycle (Ct) of qPCR shows SELECT results for detecting two m^6^A sites in 3′UTR of MCM5 in A549 cells with or without METTL3 knockdown. E,F) MCM5 levels determined by qRT‐PCR and WB assays. G) qRT‐PCR assay showing MCM5 mRNA stability. H) The effect of silencing HuR on MCM5 expression. I) Schematic diagram of wild‐type (WT) or mutant (MUT) MCM5 3′UTR fused to firefly luciferase reporter. J) Luciferase activities affected by wild‐type or mutant MCM5 3′UTR. K) RIP‐qPCR detecting binding of IGF2BP3 to the MCM5 3′UTR. L) qRT‐PCR assay showing MCM5 mRNA stability. M,N) qRT‐PCR assays showing effects of dCas13b‐ALKBH5 on MCM5 mRNA level and stability. O) Luciferase reporter assay detecting Notch signaling activities. All experiments were repeated three times with similar results. Statistical analyses were performed using a two‐tailed Student's *t*‐test, *: *p* < 0.05, **: *p* < 0.01, ***: *p* < 0.001, ****: *p* < 0.0001.

We then examined whether MCM5 is a bona fide target read by IGF2BP3. Of note, the above mentioned m^6^A seq (GSE54365) and IGF2BP3‐RIPseq (GSE90639) data showed that a specific m^6^A peak in the 3′UTR region of MCM5 mRNA coincided well with an IGF2BP3‐binding site (**Figure** **4**A). The presence of m^6^A modification in the 3′UTR region of MCM5 mRNA was confirmed by m^6^A ‐RIP‐qPCR assay (Figure 4B), and an association of IGF2BP3 with m^6^A ‐modified region of MCM5 mRNA was validated, which could be abrogated by silencing METTL3 in both A549 and HEK293FT cells (Figure 4C and Figure S4A, Supporting Information). The m^6^A modification of two potential sites (A2301 and A2309 within a DRACH motif) on the MCM5 mRNA 3′UTR were validated by SELECT‐qPCR.^[^ ^33^, ^34^ ^]^ As expected, m^6^A levels of these two nucleotide sites on MCM5 mRNA were dramatically decreased by silencing canonical m^6^A writers ((METTL3, METTL14, WTAP), whereas increased by silencing m^6^A erasers (FTO and ALKBH5) (Figure 4D and Figure S4B, Supporting Information). IGF2BP3 overexpression increased, whereas silencing IGF2BP3 reduced both mRNA and protein levels of MCM5 in LUAD cells (Figure 4E and Figure S4C, Supporting Information). Similarly, silencing METTL3 or overexpressing kinase‐dead mutant of METTL3 but not wild‐type METTL3, reversed IGF2BP3‐induced MCM5 up‐regulation (Figure 4F and Figure S4D, Supporting Information). Moreover, IGF2BP3 overexpression markedly prolonged, whereas silencing IGF2BP3 shortened, the half‐life of MCM5 mRNA; silencing mRNA stabilizer HuR dramatically diminished IGF2BP3‐induced MCM5 upregulation (Figure 4G,H). In addition, ectopic expression of IGF2BP3 significantly increased the activity of the luciferase reporter containing wild‐type, but not mutant, m^6^A ‐modified MCM5 mRNA, and the association of IGF2BP3 with m^6^A ‐modified region of MCM5 mRNA could be abrogated by introducing mutations in its putative m^6^A ‐modified site (Figure 4I–K). In parallel, the effects of IGF2BP3 on increasing MCM5 mRNA and protein levels and on promoting MCM5 mRNA stability were drastically abolished following mutating the specific m^6^A ‐modified site (Figure 4L and Figure S4E, Supporting Information). Consistently, demethylation of the m^6^A modification in MCM5 mRNA using dm^6^A CRISPR system reduced MCM5 expression and destabilized MCM5 mRNA (Figure 4M,N and Figure S4F, Supporting Information),^[^ ^35^ ^]^ and mutation of m^6^A ‐modified site in MCM5 mRNA failed to enhance IGF2BP3‐induced activation of Notch signaling (Figure 4O). Taken together, our data strongly demonstrate an essential role of IGF2BP3‐recognized m^6^A modification in upregulating MCM5 expression by promoting MCM5 mRNA stability, leading to overactivation of Notch signaling.

### 2.4 m^6^A Modified MCM5 is Crucial for IGF2BP3‐Induced Cancer Cell Plasticity to Metastasize

**Figure 5:**
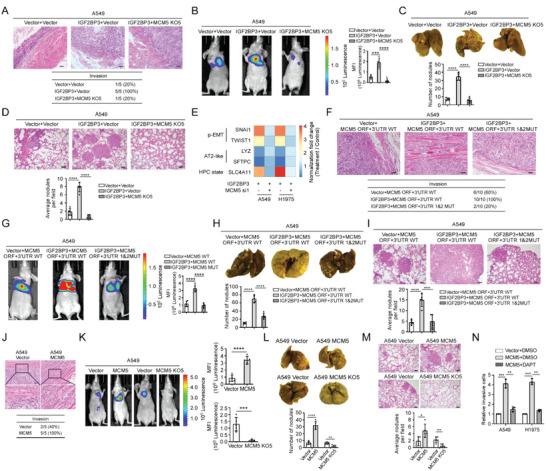
m6A modified MCM5 is crucial for IGF2BP3‐induced cancer cell plasticity. A) H&E staining shows muscle penetration in subcutaneous tumor xenografts formed by indicated cells (1 × 10^6^). *n* = 5. B–D) Bioluminescent images, picric acid staining and H&E staining show lung metastases formed by tail vein injection with indicated cells. *n* = 5. E) qRT‐PCR analysis examining p‐EMT and lineage plasticity markers. F–I) Indicated cells (1×10^6^) were injected subcutaneously (F) or intravenously (G–I), and representative bioluminescent images, picric acid staining and H&E staining of lung metastasis are shown. *n* = 5. J–M) Indicated cells (1 × 10^6^) were injected subcutaneously (J) or intravenously (K–M), and representative bioluminescent images, picric acid staining and H&E staining of lung metastases are shown. *n* = 5. Scale bars, 50 µm. N) Transwell assay showing the effect of inhibiting Notch on MCM5‐augmented invasion. For A, F, and J, the number of mice with local invasion was counted. For B–D, G, H, and K–M, the bioluminescent intensities and the number of lung surface nodules and metastatic lesions in mouse lung tissue were provided. Statistical analyses were performed using a two‐tailed Student's *t*‐test, **: *p* < 0.01, ***: *p* < 0.001, ****: *p* < 0.0001.

As expected, IGP2BP3‐potentiated migratory and invasive abilities of LUAD cells were dramatically diminished by MCM5 depletion (Figure S5A, Supporting Information). In vivo, while subcutaneous xenografts of IGF2BP3‐overexpressing A549 cells displayed marked penetration of tumor cells into neighboring subcutaneous tissue, MCM5 depletion abrogated the penetrating ability of IGF2BP3‐overexpressing A549 cells in subcutaneous xenografts (**Figure** **5**A); MCM5 depletion also greatly impaired metastatic colonization of IGF2BP3‐overexpressing A549 cells when intravenously injected (Figure 5B–D). Moreover, IGF2BP3‐induced increase in markers of p‐EMT or high‐plasticity state and decrease in markers of AT2‐like state, were remarkably reversed by MCM5 knockdown (Figure 5E). It is of particular note that the IGF2BP3‐aumented LUAD invasion, migration and metastasis were dramatically inhibited by mutating the m^6^A ‐modified site in MCM5 mRNA 3′UTR (Figure 5F–I and Figure S5B, Supporting Information), together demonstrating that the m^6^A modification on MCM5 transcript is crucial for the pro‐metastatic effects of IGF2BP3.

Additionally, similar to IGF2BP3, ectopic expression of MCM5 endowed LUAD cells with augmented abilities to migrate and invade, while knockdown of MCM5 exhibited the opposite effects (Figure S5C, Supporting Information). MCM5‐overexpressing A549 cells gained ability to penetrate into neighboring subcutaneous tissue in formed tumor xenografts and exhibited much stronger metastatic bioluminescent signals (Figure 5J–M). By contrast, MCM5‐depleted or silenced A549 cells barely formed macroscopic or microscopic lung metastases (Figure 5K–M and Figure S5D–F, Supporting Information). Notably, GSEA also revealed a positive correlation between high MCM5 level and metastasis‐related signature (Figure S5G, Supporting Information). Moreover, the pro‐invasive and pro‐migratory effects of MCM5 could be abrogated by Notch inhibitor DAPT (Figure 5N and Figure S5H, Supporting Information), further supporting that MCM5 promotes LUAD metastasis via activating Notch1 signaling.

### 2.5 MCM5 Inhibits SIRT1‐Mediated NICD1 Degradation

**Figure 6:**
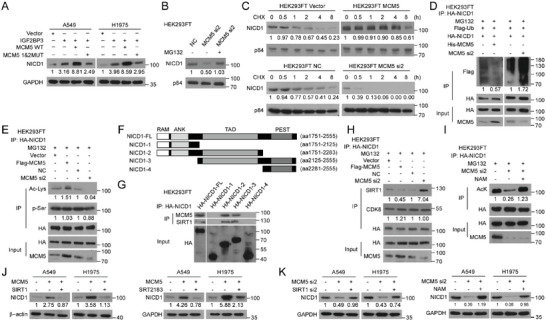
MCM5 inhibits SIRT1‐mediated NICD1 degradation. A–C) WB analysis determining NICD1 expression or NICD1 stability in indicated cells. D,E) Co‐IP assays examining the effects of MCM5 on NICD1 polyubiquitination or acetylation (AcK) in the presence of MG132. F) Schematic diagram illustrates full‐length (FL) or truncated NICD1 (NICD1‐1‐4). G) Co‐IP analysis of the interaction of MCM5 or SIRT1 with indicated NICD1 truncates. H) The effects of MCM5 on the binding between SIRT1 and NICD1. I) The reversing effect of SIRT1 inhibitor nicotinamide (NAM) on NICD1 acetylation. J,K) The effects of SIRT1 over‐expression or activation by its agonist SRT2183 (J), or SIRT1 inhibition (K) on NICD1 expression. All experiments were repeated three times with similar results.

MCM5 bound NICD1 and upregulated NICD1 protein levels without affecting NOTCH1 mRNA levels (**Figure** **6**A and Figure S6A, Supporting Information), indicating that MCM5 might prolong NICD1 stability. Indeed, proteasome inhibitor MG132 markedly abrogated the NICD1decrease induced by silencing MCM5, and overexpressing MCM5 prolonged, whereas silencing MCM5 shortened half‐lives of NICD1 proteins (Figure 6B,C). Consistently, NICD1 polyubiquitination was robustly decreased by MCM5 overexpression but increased when MCM5 was depleted (Figure 6D). CDK8‐mediated phosphorylation and SIRT1‐mediated deacetylation have been well documented for regulating NICD turnover.^[^ ^36^, ^37^ ^]^ Although without affecting NICD1 phosphorylation, MCM5 overexpression or knockdown, respectively, increased or decreased NICD1 acetylation (Figure 6E). Interestingly, both MCM5 and the deacetylase SIRT1 could bind the TAD domain of NICD1, and the binding between NICD1 and SIRT1 was impaired in MCM5‐overexpressing cells but enhanced when MCM5 was silenced (Figure 6F–H). In parallel, MCM5 knockdown‐reduced NICD1 acetylation could be blocked by a specific SIRT1 inhibitor nicotinamide (NAM) (Figure 6I). SIRT1 overexpression or activation by its agonist SRT2183 drastically inhibited MCM5‐induced NICD1 upregulation and Notch signaling activation (Figure 6J, Figure S6B,C, Supporting Information), while silencing or inhibiting SIRT1 abolished the inhibitory effects of silencing MCM5 on NICD1 levels or Notch signaling (Figure 6K, Figure S6D,E, Supporting Information). These data suggest that MCM5 competitively inhibits the binding of SIRT1 to NICD1, maintains NIDC1 acetylation and subsequently promotes NICD1 stability and Notch signaling overactivation.

### 2.6 Clinical Significance of the IGF2BP3/MCM5/Notch Axis in Metastatic LUAD

**Figure 7:**
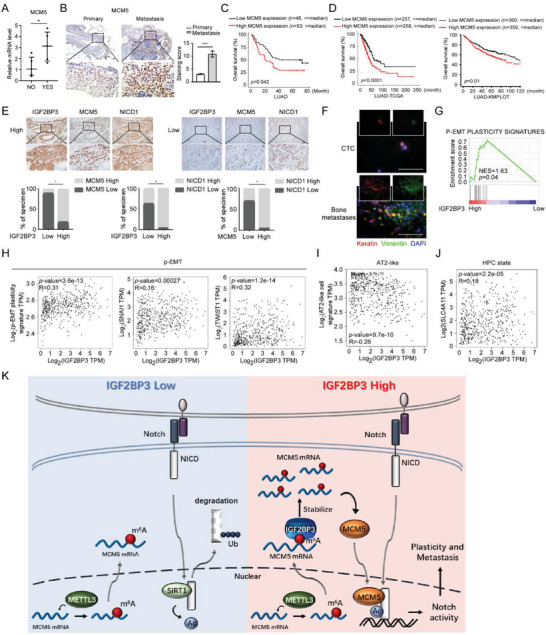
Clinical significance of the IGF2BP3/MCM5/Notch axis in metastatic LUAD. A) qRT‐PCR analysis of MCM5 mRNA in LUAD primary tumors derived from 5 non‐metastatic (NO) and 5 metastatic (YES) patients. (B) IHC of MCM5 expression in primary LUAD tumors and matched brain metastases. *n* = 10. C,D)Overall survival (OS) analysis of 99 LUAD patients in our own cohort and indicated public LUAD cohorts. E) The expression association between IGF2BP3, MCM5, and NICD1 in LUAD tissues from 50 patients. F) Analysis of epithelial (Keratin) and mesenchymal (Vimentin) markers in circulating tumor cells (CTCs) and bone metastases. Scale bar: 50 µm. G–J) Correlation analysis of IGF2BP3 and p‐EMT plasticity, AT2‐like and HPC state signatures based on the TCGA LUAD datasets using GSEA (G) or GEPIA (H–J). K) Schematic diagram of the IGF2BP3‐MCM5‐Notch axis. Scale bars, 50 µm in (B) and (E). Statistical analyses were performed using a two‐tailed Student's *t*‐test (A) and (B) or chi‐squared test (E), *: *p* < 0.05, ***: *p* < 0.001.

Similar to IGF2BP3, MCM5 upregulation was consistently observed in metastatic LUAD tumors as compared with those non‐metastatic primary tumors (**Figure** **7**A and Figure S7A, Supporting Information), and its immunostaining was much stronger in brain metastases than that in their paired primary LUAD tumors (Figure 7B). Importantly, in our cohort of 99 LUAD patients (Table S1, Supporting Information), high levels of MCM5 correlated with distant metastasis (*p* = 0.045) and clinical staging (*p* = 0.02) (Table S2, Supporting Information); LUAD patients with high‐level MCM5 expression had only 33‐month median survival time, as compared to the 48‐month median survival time of those with low‐level MCM5 (Figure 7C). Analysis of two large cohorts of public LUAD datasets showed similar results (Figure 7D and Table S3, Supporting Information). Moreover, multivariate analysis of the TCGA LUAD datasets indicate that MCM5 might represent an independent prognostic marker (Table S4, Supporting Information).

We then further explored clinical relevance of the IGF2BP3/MCM5/Notch axis in LUAD tissue with different clinical stages. Immunostaining levels of IGF2BP3, MCM5 and NICD1 were much stronger in advanced‐stage LUAD tissue than those in early‐stage LUAD tissue, whereas METTL3 expression pattern was similar in LUAD tissues with different clinical stages (Figure S7B, Supporting Information), which is in accordance with the transcriptional data from the TCGA LUAD datasets (Figure S7C, Supporting Information). Of note, similar to IGF2BP3 and MCM5, high‐level NICD1 indicated poor metastasis‐free survival of LUAD patients, whereas METTL3 expression levels were not correlated with overall or metastasis‐free survival (Figure S7D,E, Supporting Information). In a cohort of 50 LUAD clinical samples, 81.8% and 96% of the samples immunostained with strong expression of IGF2BP3, respectively, presented high levels of MCM5 and NICD1, as compared to only 11.1% and 37.5% of LUAD samples with weak IGF2BP3 staining presenting high MCM5 and NICD1 levels (Figure 7E). Consistently, there was a positive correlation between IGF2BP3 and MCM5 mRNA levels based on our analysis of the TCGA LUAD datasets (Figure S7F, Supporting Information); whereas METTL3 expression was not correlated with IGF2BP3, MCM5 or NICD in LUAD tissue (Figure S7G, Supporting Information). Impressively, circulating tumor cells (CTCs) isolated from stage IV LUAD patients with high IGF2BP3 levels displayed co‐expression of epithelial marker Keratin and mesenchymal marker Vimentin; the co‐expression of strong Keratin and Vimentin immunostaining were also detected in clinical tissues of bone metastases from LUAD patients (Figure 7F), indicating the presence of p‐EMT state in both LUAD CTCs and LUAD metastatic lesions. In addition, further analysis of IGF2BP3, MCM5, and HES1 showed that their expression positively correlated with markers of p‐EMT or high‐plasticity state, but negatively correlated with markers of AT2‐like state (Figure 7G–J, Figure S7H,I, Supporting Information). Taken together, we demonstrate that the m^6^A reader IGF2BP3 induces LUAD p‐EMT with augmented cancer cell plasticity to facilitate metastasis through recognizing m^6^A‐modified MCM5 mRNAs depending on canonical m^6^A writers such as METTL3 to stabilize them, and that high‐level MCM5 proteins bind NICD1 to competitively abrogate SIRT1‐induced deacetylation and degradation of NICD1, leading to Notch signaling overactivation (Figure 7K).

## 3 Conclusion

Although tumor cells can disseminate from primary site to distant tissue/organs via EMT program, metastasis frequently occurs via EMT‐independent mechanisms. It was reported that EMT^high^ primary tumors do not necessarily present stronger metastatic properties than EMT^low^ tumors in spontaneous breast cancer mouse models.^[^ ^38^, ^39^ ^]^ Importantly, the phenotypic changes of EMT are not readily seen even in advanced cancer patients and in clinical metastatic tumor lesions, which normally consist of non‐EMT tumor cells with epithelial features. Instead, tumor cells in this intermediate state retaining both epithelial (E) and mesenchymal (M) features can display more potent metastatic capacity.^[^ ^28^, ^40^ ^]^ However, how p‐EMT program is regulated remains largely unclear. In our current study, we demonstrate that a specific metastasis promoter IGF2BP3 induces p‐EMT and confers LUAD cells high plasticity to metastasize through overactivating the Notch1 signaling via MCM5/NICD1 axis. Inhibiting the IGF2BP3/MCM5/NICD1 axis impairs the tumor cell plasticity and potently abrogates distant metastasis of LUAD cells. Moreover, the IGF2BP3/MCM5/NICD1 axis and p‐EMT program are clinically relevant in LUAD tissues and correlate with disease progression of LUAD patients. These findings provide mechanistic insights into p‐EMT and cellular plasticity during LUAD metastasis. It might be more feasible to develop p‐EMT‐targeting anti‐cancer strategies than those directly targeting EMT.

Although m^6^A modification has been reported to play an important role in tumor metastasis,^[^ ^41^, ^42^ ^]^ how it shapes cancer cell plasticity, especially in the context of p‐EMT program, remains uncharacterized. Since m^6^A modification is dynamically and reversibly regulated by m^6^A “writers” and “erasers,” many studies have shown dual roles of m^6^A editing in tumor development and progression.^[^ ^10^, ^11^, ^12^, ^13^ ^]^ For example, the m^6^A writer METTL3 exhibits both oncogenic and tumor‐suppressive abilities in the same cancer type;^[^ ^43^ ^]^ similarly, overexpression of demethylase FTO promotes progression of breast and colon cancers, but FTO loss elicits an EMT program and promotes breast cancer metastasis.^[^ ^41^, ^44^, ^45^ ^]^ Interestingly, we find that a critical m^6^A “reader” IGF2BP3, but not m^6^A “writers” or “erasers,” is robustly upregulated in metastatic LUAD tumors. IGF2BP3 induces p‐EMT to empower LUAD cells with high plasticity to facilitate metastasis through an m^6^A‐dependent manner. IGF2BP3 recognizes m^6^A‐modified MCM5 mRNA to promote their stability and upregulate MCM5 protein levels, leading to p‐EMT‐induced cancer cell plasticity. It is of note that although our study demonstrates the essential role of METTL3‐dependent m^6^A modification in both the pro‐metastatic effects of IGF2BP3/MCM5 and Notch signaling activation, high‐level METTL3 expression in LUAD tissue is clinical stage‐independent and remains similar in metastatic versus non‐metastatic LUAD tissue, indicating that since METTL3‐written m^6^A modification globally provides fundamental regulation pattern of RNA levels especially during tumor development, the fates of m^6^A‐modified RNAs, such as RNA stability or translation efficiency, which are normally regulated by m^6^A readers, may be fairly important in determining expression levels of the m^6^A‐modified RNAs.^[^ ^46^ ^]^ Taken together, our current study identifies crucial roles of the m^6^A reader IGF2BP3 and m^6^A modification in shaping cellular plasticity and consequent tumor metastasis. Therefore, targeting m^6^A modification through inhibiting IGF2BP3 may represent a potentially valuable therapeutic strategy for treatment of LUAD metastasis.

Several reports have also shown that Notch overactivation facilitates tumor cells to undergo p‐EMT and promotes tumor metastasis.^[^ ^47^, ^48^, ^49^ ^]^ Although Notch signaling overactivation is commonly shown in metastatic tumor tissue, genetic alterations in genes involved in Notch signaling are rare in LUAD.^[^ ^18^, ^19^ ^]^ In our present study, we demonstrate a m^6^A reader IGF2BP3‐mediated mechanism through which m^6^A‐dependent epigenetic overactivation of Notch signaling is achieved in LUAD cells undergoing p‐EMT. Notably, the role of m^6^A modification on Notch activity seems context‐dependent. During zebrafish embryogenesis, m^6^A modification on notch1a mRNA could repress Notch signaling to promote hematopoietic stem and progenitor cell specification.^[^ ^50^ ^]^ In Hela cells, YTHDF2‐recognized m^6^A modification of Notch1 mRNA accelerates Notch1 mRNA degradation, whereas in esophageal cancer cells METTL3‐catalyzed m^6^A modification on Notch1 mRNA increases Notch1 expression and promotes Notch1 activation.^[^ ^20^, ^21^, ^50^, ^51^ ^]^ Interestingly, by analyzing public m^6^A MeRIP‐seq odatasets of LUAD cells (GSE54365) (Figure 3A), no specific m^6^A modifications across Notch1 transcripts are found. We show that in LUAD cells m^6^A‐modified MCM5 mRNA leads to high‐level expression of MCM5 protein, which directly binds NICD1 to competitively abrogate SIRT1‐mediated deacetylation of NICD1 and thus increases NICD1 protein stability. Of note, NICD turnover is strictly controlled to ensure optimal strength of the Notch1 signaling and previous reports mainly focus on phosphorylation regulation of NICD1 stability.^[^ ^16^, ^17^, ^36^ ^]^ The current study demonstrates the importance of MCM5/SIRT1 mediated acetylation regulation of NICD1 turnover, providing a distinct mechanistic insight in m^6^A‐dependent overactivation of Notch1 signaling in a phosphorylation‐independent manner, which should be helpful for developing Notch1 signaling‐targeted inhibitors.

## 4 Experimental Section

### Cell Culture

All tumor cell lines and human embryonic kidney cell line HEK293FT were obtained from the cell bank of Shanghai Institutes of Biological Sciences (Shanghai, China), Fu Erbo Biotechnology Co., Ltd (Guangzhou, China) or American Type Culture Collection (ATCC, Manassas, VA, USA), and maintained in DMEM medium (Invitrogen, Carlsbad, CA, USA) supplemented with 10% fetal bovine serum (HyClone, Logan, UT, USA). All LUAD cell lines were authenticated by short tandem repeat (STR) fingerprinting at the Laboratory of Forensic Medicine of Sun Yat‐sen University (Guangzhou, China), and were confirmed to be mycoplasma‐free.

### Tumor Specimens and Blood Samples from Patients

Tumor specimens of lung cancer patients diagnosed at the Sun Yat‐Sen University Cancer Center were obtained as described in our previous reports.^[^ ^52^, ^53^ ^]^ LUAD subject data and clinical samples were collected from the Integrated Traditional Chinese and Western Medicine Hospital of Southern Medical University. Matched primary or metastatic samples were obtained from the Jiangmen Central Hospital affiliated to Sun Yat‐sen University. Peripheral blood samples were obtained from 9 metastatic LUAD patients diagnosed in The First Affiliated Hospital of Sun Yat‐sen University for circulating tumor cells (CTCs) isolation. The approval number for human participants was Zhongshan Medical Ethics [2019] No. 026. All experiments were carried out with the full, informed consent of the subjects. The histological characterization and clinicopathologic staging of LUAD tissues were determined by standards provided in the newest version of Union for International Cancer Control (UICC) Tumor‐Node‐Metastasis (TNM) classification.

### RNA Extraction, Real‐Time PCR, and RNA Stability Assay

Total RNA was extracted using the TRIzol reagent (Invitrogen, Carlsbad, CA, USA) according to the manufacturer's instruction, and cDNA was synthesized on 2 µg of the total RNA template with random primers using the GoScript Reverse Transcription Mix (Promega, Madison, WI, USA). Real‐time PCR (RT‐PCR) was performed with 2×SYBR Green Master Mix (Promega, Madison, WI, USA). Expression of each gene of interest was normalized to reference gene GAPDH, and relative quantification was calculated by using 2^−ΔΔCt^ method. For RNA stability assay, indicated cells were seeded to achieve 50% confluence and subsequently treated with 10 µm actinomycin D (APExBio, Houston, TX, USA), followed by collection at indicated time points. Total RNAs were extracted and analyzed by qRT–PCR.

### Plasmids, siRNAs, and Transfection

Coding sequences of MCM5, NICD1, IGF2BP3, SIRT1, METTL3, and METTL3 MUT (aa395‐398, DPPW/APPA) proteins with tag (HA or Flag) were generated by PCR subcloning and inserted into lentiviral transfer plasmid pSin‐puro (Addgene, Cambridge, Massachusetts, USA) or retroviral transfer plasmid pQcxip‐puro (Clontech, Palo Alto, CA, USA). To deplete MCM5 or IGF2BP3 expression, specific human sgRNA sequences (sgIGF2BP3: ATATCCCGCCTCATTTACAG; sgMCM5: ATGTCGGGATTCGACGATCC), respectively, were cloned into the LentiCRISPRv2/Cas9 plasmid to generate LentiCRISPRv2/Cas9‐sgIGF2BP3 and LentiCRISPRv2/Cas9‐sgMCM5. siRNAs of IGF2BP3, METTL3, MCM5, Notch1, SIRT1, and HUR were purchased from Ribobio (Guangzhou, China), and their corresponding shRNA sequences were cloned into the pSupper‐neo plasmid (Clontech, Palo Alto, CA, USA). Transfection of plasmids or oligonucleotides was performed using the Lipofectamine 3000 reagent (Invitrogen, Carlsbad, CA, USA) according to the manufacturer's instruction.

The dCas13b‐ALKBH5 and non‐targeting gRNA plasmids were provided by Prof. Hongsheng Wang from Guangdong Key Laboratory of Chiral Molecule and Drug Discovery, School of Pharmaceutical Sciences, Sun Yat‐sen University.

### Cell Invasion and Migration Assays

As described in previous reports,^[^ ^52^ ^]^ indicated cells (3 × 10^4^) were plated on the top side of the Transwell chambers (Corning Costar Corp, Cambridge, MA, USA) coated without or with Matrigel (BD Biosciences, San Jose, CA, USA) and incubated at 37 °C for 22 h, followed by removal of cells inside the upper chamber with cotton swabs. Cells migrating or invading to the bottom side of the membrane were fixed with a mixture of methanol and acetic acid (3:1), stained with crystal violet, photographed, and quantified in 5 random fields.

### Tumor Xenografts In Vivo

All animal experimental procedures were approved by the Institutional Animal Care and Use Committee of Sun Yat‐sen University, and approval number is SYSU‐IACUC‐2019‐B619. Female BALB/c‐nu mice (6–8 weeks of age, 18–20 g) were used to investigate the pro‐invasive and pro‐metastatic effects of IGF2BP3 and MCM5. To assess local invasion, indicated cells were injected subcutaneously into the flanks of mice (1 × 10^6^ cells suspended in 100 µL sterile PBS, A549‐Vector left and A549‐IGF2BP3 or A549‐MCM5 right, *n* = 5), and the number of mice with local invasion was counted. To determine distant lung dissemination, 1 × 10^6^ indicated cells were injected into the lateral tail vein (*n* = 5), and metastases were monitored and analyzed by bioluminescent imaging assisted with Spectrum Living Image 4.0 software (Caliper Life Sciences, Hopkinton, MA, USA). Alternatively, vector control or IGF2BP3‐overexpressing A549 cells (5 × 10^5^) were intracardiacally injected. At the indicated experimental endpoints, mice were anesthetized and sacrificed, and lung tissues were fixed in picric acid containing 4% formaldehyde. Subcutaneous tumors and lung tissues were resected, sectioned (5 mm in thickness) and histologically examined by H&E staining. All the animal data had statistical analyses as presented and described.

### Immunoprecipitation and Protein Purification

Immunoprecipitation (IP) assay was performed as we previously described.^[^ ^52^, ^53^ ^]^ Lysates were prepared from 3 × 10^7^ HEK293FT cells transfected with HA‐tagged NICD1 in an NP‐40‐containing lysis buffer supplemented with protease inhibitor cocktail (Roche, Basel, Switzerland), and then immunoprecipitated with HA affinity agarose (Sigma‐Aldrich, Burlington, MA, USA) overnight at 4 °C. Beads containing affinity‐bound proteins were washed five times with immunoprecipitation wash buffer (300 mm NaCl, 10 mm HEPES pH 7.4, 0.1% NP‐40) to purify recombinant HA‐NICD1. Purification of recombinant Flag‐SIRT1 or Flag‐MCM5 was achieved by immunoprecipitation with Flag magnetic beads and subsequent elution with 3×Flag peptide, followed by incubation with HA‐NICD1. After five‐time washes with immunoprecipitation wash buffer (150 mm NaCl, 10 mm HEPES pH 7.4, 0.1% NP‐40), the precipitated protein was denatured, separated on SDS‐polyacrylamide gels and detected by WB analysis.

### Immunofluorescence Assay

Cells were seeded on coverslips in 24‐well plates and fixed with 4% paraformaldehyde. After 15 mins, cells were permeabilized with phosphate‐buffered saline (PBS) containing 0.2% Triton X‐100 (PBST) for 5 min and then blocked with 1% bovine serum albumin in PBST. Immunostaining was performed using primary antibodies, rabbit anti‐cleaved‐Notch1 (Immunoway, plano, TX, USA) and mouse anti‐MCM5 (Proteintech, Chicago, IL, USA), rabbit anti‐Keratin 5 (Abcam, Cambridge, UK) and mouse anti‐Vimentin (Huaan Biotechnology, Hangzhou, China), respectively overnight at 4 °C. After PBS washing for three times, the slides were incubated with Alexa Fluor 555 donkey anti‐rabbit IgG and Alexa Fluor 488 goat anti‐mouse IgG (Beyotime, Beijing, China) at room temperature for 1 h. The slides were counterstained with DAPI (Beyotime, Beijing, China) and images were captured using the Zeiss microscope (Carl Zeiss, Jena, Germany).

### Proximity Ligation Assay

The DuoLink In Situ Fluorescence (Sigma‐Aldrich, Burlington, MA, USA) protocol was used to perform the proximity ligation assay (PLA). Cells seeded on coverslips were fixed in PBS containing 4% paraformaldehyde for 15 min and then washed three times for 5 min with PBS. The fixed cell cultures were incubated in Duolink Blocking Solution for 1 h at 37 °C and replaced with the primary antibodies diluted in Duolink Antibody Diluent for 1 h at room temperature. The primary antibodies used for PLA included MCM5 (Proteintech, Chicago, IL, USA) and cleaved‐Notch1 (Immunoway, plano, TX, USA). The coverslips were washed twice for 5 min with Wash Buffer A, incubated with indicated PLA Probes (Sigma‐Aldrich, Burlington, MA, USA) for 1 h at 37 °C, re‐washed three times with Wash Buffer A and incubated in the ligation solution for 30 min at 37 °C, followed by another three‐time washing and incubation cycle in amplification solution for 100 min at 37 °C. The coverslips were then washed three times for 10 min in Wash Buffer B (Sigma‐Aldrich, Burlington, MA, USA) and were counterstained with DAPI (Beyotime, Beijing, China). PLA images were captured using the confocal laser scanning microscope system Olympus FV1000 (Olympus Medical Systems).

### Luciferase Reporter Assay

After seeding in triplicates in 24‐well plates and allowed to settle for 24 h, indicated cells were transfected with 200 ng signaling luciferase reporter (OriGene, Rockwell, Maryland, USA) plus 5 ng pRL‐TK renilla reporter plasmid (Promega, Madison, WI, USA), and siRNA (siNC or siMCM5) or pGL3‐MCM5‐3′UTR (wild‐type or mutant) plasmid by using the Lipofectamine 3000 reagent (Invitrogen, Carlsbad, CA, USA). Dual‐Luciferase reporter assays were performed after 48 h transfection according to the manufacturer's protocol using the Dual Luciferase Reporter Assay Kit (Promega, Madison, WI, USA).

### RNA Immunoprecipitation and MeRIP‐qPCR

Indicated cells were crosslinked and harvested for RIP by using anti‐Flag magnetic beads or protein‐G beads (Sigma‐Aldrich, Burlington, MA, USA). Input and co‐immunoprecipitated RNA was extracted using the Magna RIP RNA‐Binding Protein Immunoprecipitation Kit (Millipore, Bedford, MA, USA), and the extract was analyzed by qPCR. Subsequently, MeRIP was performed on an Illumina Novaseq 6000 platform commercially by the LC‐BIO Bio‐tech Ltd. (Hangzhou, China). Fifty microgram of total RNA was subject to isolation of Poly (A) mRNA using poly‐T oligonucleotide‐attached magnetic beads (Invitrogen, Carlsbad, CA, USA). After purification, the mRNA fraction was chemically fragmented into ≈100 nt‐long oligonucleotides, followed by incubation with m^6^A‐specific antibody (Synaptic Systems, Germany) in IP buffer (50 mm Tris‐HCl, 750 mm NaCl and 0.5% Igepal CA‐630) supplemented with BSA (0.5 µg µL^−1^). The mixture was then incubated with protein‐A beads and eluted with elution buffer (1 × IP buffer and 6.7 mm m^6^A). Eluted RNA was precipitated by 75% ethanol. Enrichment of m^6^A‐modified mRNAs was analyzed with qRT‐PCR.

### SELECT‐qPCR

SELECT was performed based on qPCR amplification of single‐base elongation and ligation, during which m^6^A modification hinders the single‐base elongation activity of DNA polymerases and the nick ligation efficiency of DNA ligases.^[^ ^33^, ^34^ ^]^ Total RNA was extracted using the TRIzol reagent (Invitrogen, Carlsbad, CA, USA) according to the manufacturer's instruction, and 1 µg total RNA of indicated cells was mixed with 40 nm upstream and downstream primers and 5 µm dNTP in the CutSmart buffer (New England Biolabs, Harrisburg, PA, USA). The mixture was incubated with the following program: 90 °C for 1 min, 80 °C for 1 min, 70 °C for 1 min, 60 °C for 1 min, 50 °C for 1 min, and 40 °C for 6 min. The sample was further mixed with 0.5 U SplintR ligase (New England Biolabs, Harrisburg, PA, USA), 10 nm ATP (Sangong Bioengineering, Shanghai, China) and 3 µL of 0.01 U Bst 2.0 DNA polymerase (New England Biolabs, Harrisburg, PA, USA) and incubated at 40 °C for 20 min and denatured at 80 °C for 20 min. Subsequently, the product was analyzed by qPCR with the following program: 95 °C, 5 min; (95 °C, 10 s; 60 °C, 35 s) × 40 cycles; 95 °C, 15 s; 60 °C, 1 min; 95 °C, 15 s; 4 °C held. Results were calculated by normalizing the Ct values of the samples to their corresponding Ct values of control.

### Western Blotting Analysis

Western blotting analysis was performed according to a standard method previously described by using the following antibodies: anti‐MCM5 (Proteintech, Chicago, IL, USA),^[^ ^52^, ^53^ ^]^ and anti‐IGF2BP3 (Abcam, Cambridge, UK), anti‐Flag and anti‐HA (Cell Signaling, Danvers, USA), anti‐ cleaved‐Notch1, and anti‐SIRT1 (Immunoway, plano, TX, USA), respectively. Blotted membranes were stripped and re‐blotted with an anti‐p84 rabbit monoclonal antibody (Sigma, St. Louis, MO, USA) or anti‐*β*‐actin rabbit monoclonal antibody (Sigma, Saint Louis, MO, USA) as loading controls.

### Immunohistochemistry Assays

Paraffin‐embedded LUAD sections were immunostained with antibodies against MCM5 (Abcam, Cambridge, UK, 1:200), cleaved‐Notch1 (Immunoway, plano, TX, USA, 1:200), IGF2BP3 (Abcam, Cambridge, UK, 1:150), and METTL3 (Huaan Biotechnology, Hangzhou, China, 1:100), respectively. The degree of immunostaining of indicated proteins was evaluated and scored by two independent observers with both the proportions of positively stained tumor cells and the staining intensities. Scores representing the proportion of positively stained tumor cells was graded as: 0 (no positive tumor cells), 1 (<5%), 2 (5%–25%), 3 (25%–50%), and 4 (>50%). The intensity of staining was determined as: 0 (no staining); 1 (weak staining = light yellow), 2 (moderate staining = yellow brown), and 3 (strong staining = brown). The staining index (SI) was calculated as the function of staining intensity × percentage of positive tumor cells, resulting in scores of 0, 1, 2, 3, 4, 6, 8, 9, and 12. Cutoff values for high‐ and low‐expression of protein were chosen based on a measurement of heterogeneity using the log‐rank test with respect to overall survival. The optimal cutoff was identified as: the SI score ≥4 was considered as high expression, and ≤3 as low expression. Chi‐squared (*χ* ^2^) tests were used for contingency tables.

### CTCs Isolation and CTCs Immunostaining

CTCs isolation was performed using negative enrichment approach according to the instruction of Guangzhou Xinde Pharmaceutical Company. Peripheral blood (5 mL) from metastatic LUAD patients was collected in a tube with acid citrate dextrose (ACD) anticoagulant (containing 0.8 mL of anticoagulant), and CTCs isolation was performed by using a CEP8 amplificated CTC detection kit (Cyttel, Cyttel Bio, China). In brief, 5 mL of peripheral blood were transferred into 50 mL tubes containing 40 mL CS1 working solution after thorough mixing. Subsequently, the solution was centrifuged at 500 g for 5 min at room temperature. Supernatants were discarded and CS2 working buffer was added into the tubes to remove the red blood cells. CS3 working buffer and magnetic beads were subsequently used to deplete the majority (>99.9%) of leukocytes by magnetic separation and gradient centrifugation. The middle cell layer was transferred to another clean tube for further centrifugation. Following centrifugation, the upper liquid (100 µL) was discarded and 200 µL CF1 stationary liquid was added to resuspend the remaining cells. Finally, the cells were applied onto coated CTC PEN membrane slides and dried overnight at room temperature for immediate immunofluorescence staining of epithelial and mesenchymal markers by corresponding primary antibodies, namely, rabbit anti‐Keratin 5 (Abcam, Cambridge, UK) and mouse anti‐Vimentin (Huaan Biotechnology, Hangzhou, China), respectively. The slides were counterstained with DAPI (Beyotime, Beijing, China) and images were captured using the Zeiss microscope (Carl Zeiss, Jena, Germany).

### Public Sequencing Datasets and Data Analysis

The RIP‐seq (GSE90639), MeRIP‐seq (GSE29714; GSE54365), and RNA‐seq (GSE90684; GSE126548) databases were obtained from the public database Gene Expression Omnibus (GEO) of National Center for Biotechnology Information (NCBI) website. For these sequencing data, low quality read ends and remaining parts of sequencing adapters were trimmed by using Cutadapt v1.15.^[^ ^54^ ^]^ The processed reads were mapped to human genome version hg19 by HISAT2 v2.1.0 with default settings.^[^ ^55^ ^]^ Differential gene expression was calculated by Cuffdiff v2.2.1.^[^ ^56^ ^]^ For the RIP‐seq, the RIP targets were defined as genes with RPKM (reads per kilobase, per million reads) values ≥1, immunoprecipitation/input ratio ≥2, and *p* < 0.05. For the m^6^A‐seq experiment, after trimming and mapping, confident m^6^A peaks were calculated by exomePeak v2.14.0 with immunoprecipitation/input ratio ≥2 and *p* < 0.01.^[^ ^57^ ^]^ For the RNA‐seq, raw counts were generated by htseq v0.11.2.^[^ ^58^ ^]^ Differentially expressed genes were analyzed by DESeq2 v1.28.1.^[^ ^59^ ^]^

### RNA Sequencing and Analysis

Total RNA was isolated from METTL3‐knocked down or vector‐control A549 cells using TRIzol (Invitrogen, Carlsbad, CA, USA) and were subjected to RNA sequencing by the LC‐BIO Bio‐tech Ltd. (Hangzhou, China). Reads were mapped to human genome version hg19 by HISAT2 version 2.1.0 with default settings, and raw counts were generated by htseq v0.11.2. Differential gene expression was calculated by R package DESeq2 v1.28.1. The volcano map of the differentially expressed genes was produced by the R package ggplot2 v3.3.0. These RNA‐seq data were deposited in the NCBI GEO (http://www.ncbi.nlm.nih.gov/geo/), with accession number GSE211425.

### Gene Set Enrichment Analysis

The Cancer Genome Atlas (TCGA) LUAD datasets were downloaded from dataset portal (https://www.tcga‐data.nci.nih.gov/). GSEA software (version 4.0.2, http://www.broadinstitute.org/gsea) was used to identify the association of metastasis‐related or Notch‐related gene signatures with MCM5 or IGF2BP3. The normalized enrichment score (NES) and nominal *p*‐value were calculated for comparison. Bioinformatic analysis for visual heatmap was performed using the MeV 4.4 program.

### Data Availability 

The raw data and processed data of single‐cell RNA‐sequencing data and single‐cell VDJ‐sequencing data were deposited in the Gene Expression Omnibus (GEO) database under accession code GSE167036. The raw data and processed data of spatial transcriptomic data generated in this study were deposited in the Gene Expression Omnibus (GEO) database under accession code GSE190811. The publicly available single cell dataset used in this study were available from the Gene Expression Omnibus (accession numbers GSE11472712). Source data are provided in this paper as a Source data file. The remaining data are available within the Article, Supplementary Information, or Source Data file. Source data are provided with this paper.

### Study Approval

The study was approved by the Medical Ethics Committee of the Sun Yat‐Sen University. For in vivo tumor experiments, all procedures were approved by the Institutional Animal Care and Use Committees of Sun Yat‐Sen University.

### Statistical Analysis

All statistical analyses were performed using the SPSS 20.0 (International Business Machines Corporation, Armonk, NY, USA) statistical software package. For clinical survival analysis, LUAD patients were divided into two groups according to median value of IGF2BP3, MCM5, METTL3, or NICD1 expression and were comparatively analyzed by the Kaplan–Meier method with a log rank test. Univariate and multivariable survival analysis were performed using Cox regression analysis. Correlations were analyzed by using Pearson's correlation. Comparisons between groups for statistical significance were performed with a two‐tailed Student's *t*‐test. Error bars represent mean ± SD derived from three independent experiments. In all cases, *p* value < 0.05 was considered statistically significant. All experiments were performed for at least three times independently under similar conditions, unless otherwise specified in the figure captions.

## Conflict of Interest

The authors declare no conflict of interest.

## Author Contributions

X.Y. And Q.B. contributed equally to this work. Y.Y. and J.C. contributed conceptualization. Y.Y. contributed project administration. J.W., J.L., Z.C., and A.Z. contributed methodology. X.Y., Q.B., W.C., H.T., J.L., F.W., W.G., Q.L., X.D., and N.Z. contributed experiments. Q.B. contributed analyses of sequencing data. M.L. provided supervision. Y.Y. contributed writing of original draft. J.C. and M.L. contributed review and editing of the manuscript.

## Supporting information

Supporting Information

## Data Availability

The data that support the findings of this study are available on request from the corresponding author. The data are not publicly available due to privacy or ethical restrictions.

## References

[1] D. Consonni , M. Pierobon , M. H. Gail , M. Rubagotti , M. Rotunno , A. Goldstein , L. Goldin , J. Lubin , S. Wacholder , N. E. Caporaso , P. A. Bertazzi , M. A. Tucker , A. C. Pesatori , M. T. Landi , J. Natl. Cancer Inst. 2015, 107, djv059. 25802059 10.1093/jnci/djv059 PMC4838060

[2] M. Riihimaki , A. Hemminki , M. Fallah , H. Thomsen , K. Sundquist , J. Sundquist , K. Hemminki , Lung Cancer 2014, 86, 78. 25130083 10.1016/j.lungcan.2014.07.020

[3] S. Yuan , R. J. Norgard , B. Z. Stanger , Cancer Discov. 2019, 9, 837. 30992279 10.1158/2159-8290.CD-19-0015 PMC6606363

[4] M. A. Nieto , Science 2013, 342, 1234850. 24202173 10.1126/science.1234850

[5] A. Dongre , R. A. Weinberg , Nat. Rev. Mol. Cell Biol. 2019, 20, 69. 30459476 10.1038/s41580-018-0080-4

[6] A. W. Lambert , D. R. Pattabiraman , R. A. Weinberg , Cell 2017, 168, 670. 28187288 10.1016/j.cell.2016.11.037 PMC5308465

[7] L. M. LaFave , V. K. Kartha , S. Ma , K. Meli , I. Del Priore , C. Lareau , S. Naranjo , P. M. K. Westcott , F. M. Duarte , V. Sankar , Z. Chiang , A. Brack , T. Law , H. Hauck , A. Okimoto , A. Regev , J. D. Buenrostro , T. Jacks , Cancer Cell 2020, 38, 212. 32707078 10.1016/j.ccell.2020.06.006 PMC7641015

[8] H. Huang , H. Weng , J. Chen , Cancer Cell 2020, 37, 270. 32183948 10.1016/j.ccell.2020.02.004 PMC7141420

[9] S. Zaccara , R. J. Ries , S. R. Jaffrey , Nat. Rev. Mol. Cell Biol. 2019, 20, 608. 31520073 10.1038/s41580-019-0168-5

[10] Y. Wang , M. Li , L. Zhang , Y. Chen , S. Zhang , Mol. Ther. Oncolytics 2021, 21, 367. 34169146 10.1016/j.omto.2021.04.011 PMC8190133

[11] D. Jin , J. Guo , Y. Wu , L. Yang , X. Wang , J. Du , J. Dai , W. Chen , K. Gong , S. Miao , X. Li , H. Sun , Mol. Cancer 2020, 19, 40. 32106857 10.1186/s12943-020-01161-1 PMC7045432

[12] D. Jin , J. Guo , Y. Wu , J. Du , L. Yang , X. Wang , W. Di , B. Hu , J. An , L. Kong , L. Pan , G. Su , J. Hematol. Oncol. 2019, 12, 135. 31818312 10.1186/s13045-019-0830-6 PMC6902496

[13] F. Li , J. Zhao , L. Wang , Y. Chi , X. Huang , W. Liu , Mol. Biotechnol. 2022, 64, 199. 34586620 10.1007/s12033-021-00406-8

[14] Y. Yang , Y. H. Ahn , D. L. Gibbons , Y. Zang , W. Lin , N. Thilaganathan , C. A. Alvarez , D. C. Moreira , C. J. Creighton , P. A. Gregory , G. J. Goodall , J. M. Kurie , J. Clin. Invest. 2011, 121, 1373. 21403400 10.1172/JCI42579 PMC3069760

[15] J. Choi , Y. J. Jang , C. Dabrowska , E. Iich , K. V. Evans , H. Hall , S. M. Janes , B. D. Simons , B. K. Koo , J. Kim , J. H. Lee , Nat. Cell Biol. 2021, 23, 953. 34475534 10.1038/s41556-021-00742-6 PMC7611842

[16] R. Kopan , M. X. Ilagan , Cell 2009, 137, 216. 19379690 10.1016/j.cell.2009.03.045 PMC2827930

[17] E. R. Andersson , U. Lendahl , Nat. Rev. Drug Discov. 2014, 13, 357. 24781550 10.1038/nrd4252

[18] D. J. H. Shih , N. Nayyar , I. Bihun , I. Dagogo‐Jack , C. M. Gill , E. Aquilanti , M. Bertalan , A. Kaplan , M. R. D'Andrea , U. Chukwueke , F. M. Ippen , C. Alvarez‐Breckenridge , N. D. Camarda , M. Lastrapes , D. McCabe , B. Kuter , B. Kaufman , M. R. Strickland , J. C. Martinez‐Gutierrez , D. Nagabhushan , M. De Sauvage , M. D. White , B. A. Castro , K. Hoang , A. Kaneb , E. D. Batchelor , S. H. Paek , S. H. Park , M. Martinez‐Lage , A. S. Berghoff , et al., Nat. Genet. 2020, 52, 371. 32203465 10.1038/s41588-020-0592-7 PMC7136154

[19] J. Chen , H. Yang , A. S. M. Teo , L. B. Amer , F. G. Sherbaf , C. Q. Tan , J. J. S. Alvarez , B. Lu , J. Q. Lim , A. Takano , R. Nahar , Y. Y. Lee , C. Z. J. Phua , K. P. Chua , L. Suteja , P. J. Chen , M. M. Chang , T. P. T. Koh , B. H. Ong , D. Anantham , A. A. L. Hsu , A. Gogna , C. W. Too , Z. W. Aung , Y. F. Lee , L. Wang , T. K. H. Lim , A. Wilm , P. S. Choi , P. Y. Ng , et al., Nat. Genet. 2020, 52, 177. 32015526 10.1038/s41588-019-0569-6

[20] J. Lv , Y. Zhang , S. Gao , C. Zhang , Y. Chen , W. Li , Y. G. Yang , Q. Zhou , F. Liu , Cell Res. 2018, 28, 249. 29148543 10.1038/cr.2017.143 PMC5799811

[21] B. Lee , S. Lee , J. Shim , Int. J. Biol. Sci. 2021, 17, 3776. 34671198 10.7150/ijbs.61573 PMC8495403

[22] X. Q. Ge , D. A. Jackson , J. J. Blow , Genes Dev. 2007, 21, 3331. 18079179 10.1101/gad.457807 PMC2113033

[23] J. W. Semple , B. P. Duncker , Biotechnol. Adv. 2004, 22, 621. 15364349 10.1016/j.biotechadv.2004.06.001

[24] N. Kim , H. K. Kim , K. Lee , Y. Hong , J. H. Cho , J. W. Choi , J.‐I. Lee , Y.‐L. Suh , B. M. Ku , H. H. Eum , S. Choi , Y.‐L. Choi , J.‐G. Joung , W.‐Y. Park , H. A. Jung , J.‐M. Sun , S.‐H. Lee , J. S. Ahn , K. Park , M.‐J. Ahn , H.‐O. Lee , Nat. Commun. 2020, 11, 2285. 32385277 10.1038/s41467-020-16164-1 PMC7210975

[25] D. Lambrechts , E. Wauters , B. Boeckx , S. Aibar , D. Nittner , O. Burton , A. Bassez , H. Decaluwé , A. Pircher , K. Van den Eynde , B. Weynand , E. Verbeken , P. De Leyn , A. Liston , J. Vansteenkiste , P. Carmeliet , S. Aerts , B. Thienpont , Nat. Med. 2018, 24, 1277. 29988129 10.1038/s41591-018-0096-5

[26] C. Kroger , A. Afeyan , J. Mraz , E. N. Eaton , F. Reinhardt , Y. L. Khodor , P. Thiru , B. Bierie , X. Ye , C. B. Burge , R. A. Weinberg , Proc. Natl. Acad. Sci. U. S. A. 2019, 116, 7353. 30910979 10.1073/pnas.1812876116 PMC6462070

[27] Y. Xu , D. K. Lee , Z. Feng , Y. Xu , W. Bu , Y. Li , L. Liao , J. Xu , Proc. Natl. Acad. Sci. U. S. A. 2017, 114, 11494. 29073077 10.1073/pnas.1618091114 PMC5664488

[28] S. V. Puram , I. Tirosh , A. S. Parikh , A. P. Patel , K. Yizhak , S. Gillespie , C. Rodman , C. L. Luo , E. A. Mroz , K. S. Emerick , D. G. Deschler , M. A. Varvares , R. Mylvaganam , O. Rozenblatt‐Rosen , J. W. Rocco , W. C. Faquin , D. T. Lin , A. Regev , B. E. Bernstein , Cell 2017, 171, 1611. 29198524 10.1016/j.cell.2017.10.044 PMC5878932

[29] D. Yang , M. G. Jones , S. Naranjo , W. M. Rideout 3rd , K. H. J. Min , R. Ho , W. Wu , J. M. Replogle , J. L. Page , J. J. Quinn , F. Horns , X. Qiu , M. Z. Chen , W. A. Freed‐Pastor , C. S. McGinnis , D. M. Patterson , Z. J. Gartner , E. D. Chow , T. G. Bivona , M. M. Chan , N. Yosef , T. Jacks , J. S. Weissman , Cell 2022, 185, 1905. 35523183 10.1016/j.cell.2022.04.015 PMC9452598

[30] N. D. Marjanovic , M. Hofree , J. E. Chan , D. Canner , K. Wu , M. Trakala , G. G. Hartmann , O. C. Smith , J. Y. Kim , K. V. Evans , A. Hudson , O. Ashenberg , C. B. M. Porter , A. Bejnood , A. Subramanian , K. Pitter , Y. Yan , T. Delorey , D. R. Phillips , N. Shah , O. Chaudhary , A. Tsankov , T. Hollmann , N. Rekhtman , P. P. Massion , J. T. Poirier , L. Mazutis , R. Li , J. H. Lee , A. Amon , et al., Cancer Cell 2020, 38, 229. 32707077 10.1016/j.ccell.2020.06.012 PMC7745838

[31] Y. Yang , P. J. Hsu , Y. S. Chen , Y. G. Yang , Cell Res. 2018, 28, 616. 29789545 10.1038/s41422-018-0040-8 PMC5993786

[32] A. Yatim , C. Benne , B. Sobhian , S. Laurent‐Chabalier , O. Deas , J. G. Judde , J. D. Lelievre , Y. Levy , M. Benkirane , Mol. Cell 2012, 48, 445. 23022380 10.1016/j.molcel.2012.08.022 PMC3595990

[33] Y. Xiao , Y. Wang , Q. Tang , L. Wei , X. Zhang , G. Jia , Angew. Chem., Int. Ed. Engl. 2018, 57, 15995. 30345651 10.1002/anie.201807942

[34] Z. Li , Y. Peng , J. Li , Z. Chen , F. Chen , J. Tu , S. Lin , H. Wang , Nat. Commun. 2020, 11, 2578. 32444598 10.1038/s41467-020-16306-5 PMC7244544

[35] J. Li , Z. Chen , F. Chen , G. Xie , Y. Ling , Y. Peng , Y. Lin , N. Luo , C. M. Chiang , H. Wang , Nucleic Acids Res. 2020, 48, 5684. 32356894 10.1093/nar/gkaa269 PMC7261189

[36] C. J. Fryer , J. B. White , K. A. Jones , Mol. Cell 2004, 16, 509. 15546612 10.1016/j.molcel.2004.10.014

[37] V. Guarani , G. Deflorian , C. A. Franco , M. Kruger , L. K. Phng , K. Bentley , L. Toussaint , F. Dequiedt , R. Mostoslavsky , M. H. H. Schmidt , B. Zimmermann , R. P. Brandes , M. Mione , C. H. Westphal , T. Braun , A. M. Zeiher , H. Gerhardt , S. Dimmeler , M. Potente , Nature 2011, 473, 234. 21499261 10.1038/nature09917 PMC4598045

[38] A. J. Trimboli , K. Fukino , A. de Bruin , G. Wei , L. Shen , S. M. Tanner , N. Creasap , T. J. Rosol , M. L. Robinson , C. Eng , M. C. Ostrowski , G. Leone , Cancer Res. 2008, 68, 937. 18245497 10.1158/0008-5472.CAN-07-2148

[39] E. D. Williams , D. Gao , A. Redfern , E. W. Thompson , Nat. Rev. Cancer 2019, 19, 716. 31666716 10.1038/s41568-019-0213-x PMC7055151

[40] B. Bakir , A. M. Chiarella , J. R. Pitarresi , A. K. Rustgi , Trends Cell Biol. 2020, 30, 764. 32800658 10.1016/j.tcb.2020.07.003 PMC7647095

[41] J. Jeschke , E. Collignon , C. Al Wardi , M. Krayem , M. Bizet , Y. Jia , S. Garaud , Z. Wimana , E. Calonne , B. Hassabi , R. Morandini , R. Deplus , P. Putmans , G. Dube , N. K. Singh , A. Koch , K. Shostak , L. Rizzotto , R. L. Ross , C. Desmedt , Y. Bareche , F. Rothe , J. Lehmann‐Che , M. Duterque‐Coquillaud , X. Leroy , G. Menschaert , L. Teixeira , M. Guo , P. A. Limbach , P. Close , et al., Nat Cancer 2021, 2, 611. 35121941 10.1038/s43018-021-00223-7 PMC10734094

[42] G. Chang , L. Shi , Y. Ye , H. Shi , L. Zeng , S. Tiwary , J. T. Huse , L. Huo , L. Ma , Y. Ma , S. Zhang , J. Zhu , V. Xie , P. Li , L. Han , C. He , S. Huang , Cancer Cell 2020, 38, 857. 33125861 10.1016/j.ccell.2020.10.004 PMC7738369

[43] Z. Zhou , J. Lv , H. Yu , J. Han , X. Yang , D. Feng , Q. Wu , B. Yuan , Q. Lu , H. Yang , Mol. Cancer 2020, 19, 104. 32513173 10.1186/s12943-020-01216-3 PMC7278081

[44] Y. Niu , Z. Lin , A. Wan , H. Chen , H. Liang , L. Sun , Y. Wang , X. Li , X. F. Xiong , B. Wei , X. Wu , G. Wan , Mol. Cancer 2019, 18, 46. 30922314 10.1186/s12943-019-1004-4 PMC6437932

[45] J. Wang , Y. Qiao , M. Sun , H. Sun , F. Xie , H. Chang , Y. Wang , J. Song , S. Lai , C. Yang , X. Li , S. Liu , X. Zhao , K. Ni , K. Meng , S. Zhang , C. Shan , C. Zhang , Clin. Transl. Med. 2022, 12, e772. 35297218 10.1002/ctm2.772 PMC8926902

[46] I. Barbieri , T. Kouzarides , Nat. Rev. Cancer 2020, 20, 303. 32300195 10.1038/s41568-020-0253-2

[47] M. Boareto , M. K. Jolly , A. Goldman , M. Pietila , S. A. Mani , S. Sengupta , E. Ben‐Jacob , H. Levine , J. N. Onuchic , J. R. Soc. Interface 2016, 13, 20151106. 27170649 10.1098/rsif.2015.1106 PMC4892257

[48] F. Bocci , M. K. Jolly , S. C. Tripathi , M. Aguilar , S. M. Hanash , H. Levine , J. N. Onuchic , J. R. Soc. Interface 2017, 14, 20170512. 29187638 10.1098/rsif.2017.0512 PMC5721160

[49] C. Liao , Q. Wang , J. An , Q. Long , H. Wang , M. Xiang , M. Xiang , Y. Zhao , Y. Liu , J. Liu , X. Guan , Int. J. Biol. Sci. 2021, 17, 3036. 34421348 10.7150/ijbs.61566 PMC8375241

[50] C. Zhang , Y. Chen , B. Sun , L. Wang , Y. Yang , D. Ma , J. Lv , J. Heng , Y. Ding , Y. Xue , X. Lu , W. Xiao , Y. G. Yang , F. Liu , Nature 2017, 549, 273. 28869969 10.1038/nature23883

[51] H. Han , C. Yang , S. Zhang , M. Cheng , S. Guo , Y. Zhu , J. Ma , Y. Liang , L. Wang , S. Zheng , Z. Wang , D. Chen , Y. Z. Jiang , S. Lin , Mol. Ther. Nucleic Acids 2021, 26, 333. 34513313 10.1016/j.omtn.2021.07.007 PMC8416973

[52] Y. Yang , L. Liu , Y. Zhang , H. Guan , J. Wu , X. Zhu , J. Yuan , M. Li , Int. J. Cancer 2014, 135, 1531. 24550137 10.1002/ijc.28799

[53] L. Liu , Y. Yang , S. Liu , T. Tao , J. Cai , J. Wu , H. Guan , X. Zhu , Z. He , J. Li , E. Song , M. Zeng , M. Li , Oncogene 2019, 38, 747. 30177836 10.1038/s41388-018-0473-z PMC6355651

[54] A. Kechin , U. Boyarskikh , A. Kel , M. Filipenko , J. Comput. Biol. 2017, 24, 1138. 28715235 10.1089/cmb.2017.0096

[55] D. Kim , B. Langmead , S. L. Salzberg , Nat. Methods 2015, 12, 357. 25751142 10.1038/nmeth.3317 PMC4655817

[56] C. Trapnell , B. A. Williams , G. Pertea , A. Mortazavi , G. Kwan , M. J. van Baren , S. L. Salzberg , B. J. Wold , L. Pachter , Nat. Biotechnol. 2010, 28, 511. 20436464 10.1038/nbt.1621 PMC3146043

[57] J. Meng , Z. Lu , H. Liu , L. Zhang , S. Zhang , Y. Chen , M. K. Rao , Y. Huang , Methods 2014, 69, 274. 24979058 10.1016/j.ymeth.2014.06.008 PMC4194139

[58] S. Anders , P. T. Pyl , W. Huber , Bioinformatics 2015, 31, 166. 25260700 10.1093/bioinformatics/btu638 PMC4287950

[59] M. I. Love , W. Huber , S. Anders , Genome Biol. 2014, 15, 550. 25516281 10.1186/s13059-014-0550-8 PMC4302049

