# Supplementary material for: m6A‐Dependent Modulation via IGF2BP3/MCM5/Notch Axis Promotes Partial EMT and LUAD Metastasis
Source: Adv Sci (Weinh). 2023 May 12;10(20):2206744. doi: 10.1002/advs.202206744 (PMC10369244; doi:10.1002/advs.202206744)
Supplement: Supplementary file 1 — Supporting Information [file ADVS-10-2206744-s001.pdf]

ADVANCED SCIENCE
Open Access

Supporting Information

for *Adv. Sci.*, DOI 10.1002/advs.202206744

m^{6}A-Dependent Modulation via IGF2BP3/MCM5/Notch Axis Promotes Partial EMT and LUAD Metastasis

Xia Yang, Qiaorui Bai, Weizhong Chen, Jiaer Liang, Fang Wang, Weiqi Gu, Lei Liu, Quanfeng Li, Zishuo Chen, Anni Zhou, Jianting Long, Han Tian, Jueheng Wu, Xiaofan Ding, Ningning Zhou, Mengfeng Li, Yi Yang and Junchao Cai**

m^{6}A-dependent modulation via IGF2BP3/MCM5/Notch axis promotes partial EMT and LUAD metastasis.

Xia Yang[1,#], Qiaorui Bai[1,#], Weizhong Chen[2], Jiaer Liang[1], Fang Wang[1], Weiqi Gu[1], Lei Liu[3], Quanfeng Li[4], Zishuo Chen[5], Anni Zhou[2], Jianting Long[6], Han Tian[1], Jueheng Wu[1], Xiaofan Ding[7], Ningning Zhou[8], Mengfeng Li[1,5], Yi Yang[2,], Junchao Cai[1,9]**

Supplementary figure 1.

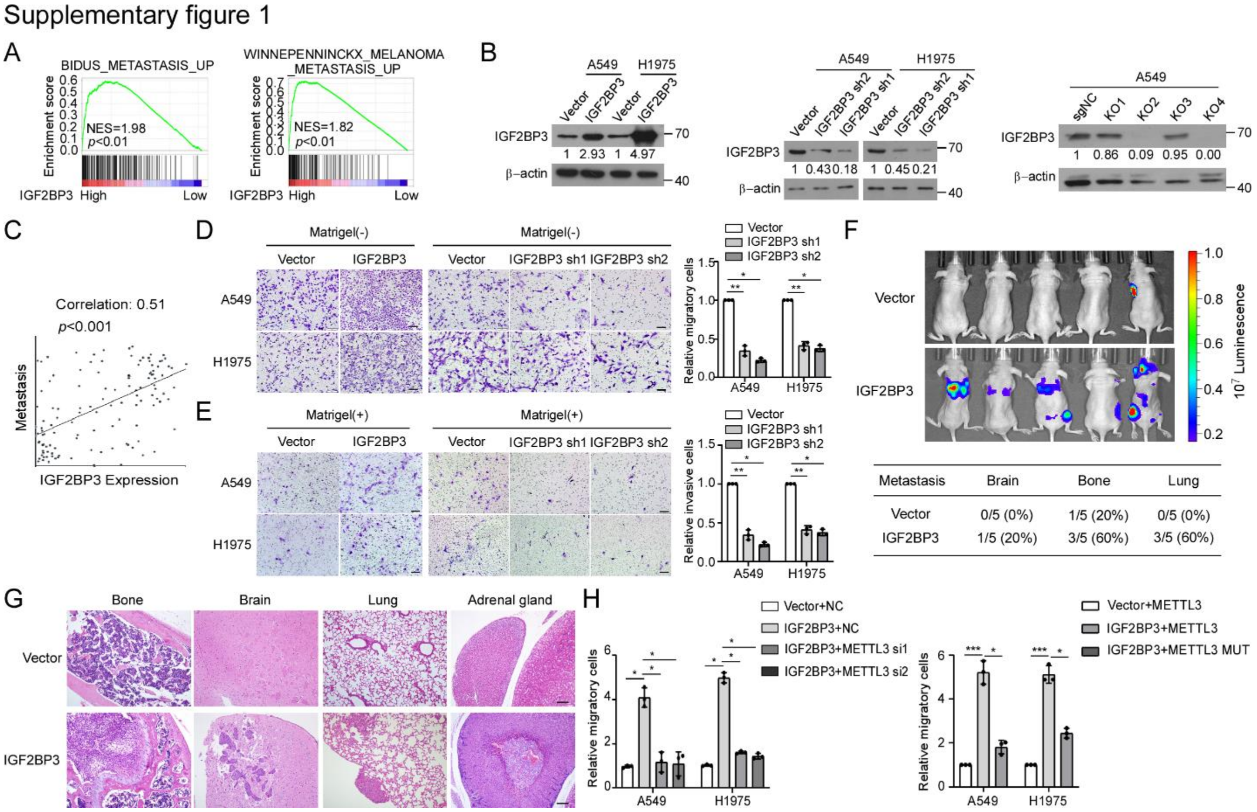

Metastasis	Brain	Bone	Lung
Vector	0/5 (0%)	1/5 (20%)	0/5 (0%)
IGF2BP3	1/5 (20%)	3/5 (60%)	3/5 (60%)

(A) Gene Set Enrichment Analysis (GSEA) of the TCGA-LUAD datasets showing correlation between IGF2BP3 expression and tumor metastasis-related signatures. **(B)** Expression efficiencies of IGF2BP3 stably overexpressed, knocked down and knocked out cell lines. Representative images from three independent experiments are shown. **(C)** Correlation of metastasis signature and IGF2BP3 expression from the CancerSEA LUAD database. (**D)** and **(E)** IGF2BP3-overexpressing or -silenced LUAD cells were plated in chambers without **(D)** or with **(E)** matrigel to indicate metastasis-related traits in transwell assays. Migratory or invasive cells were quantified in five random fields. **(F)** Indicated A549 cells (5×10^5) were injected into the

cardiac ventricle of nude mice. Representative bioluminescent images of systemic metastasis and counts of metastases in various organs are shown, n=5. **(G)** Organs were dissected 28 days after injection of tumor cells, and indicated group of H&E stained organs are shown. Scale bars, 50 μm. **(H)** Transwell assays showing metastasis-related traits affected by indicated treatment. Migratory cells were quantified in five random fields. Error bars represent the means ± SD derived from three independent experiments. Statistical analyses were performed using a two-tailed Student's t-test, *: $P < 0.05$, **: $P < 0.01$, ***: $P < 0.001$.

Supplementary figure 2.

Supplementary figure 2

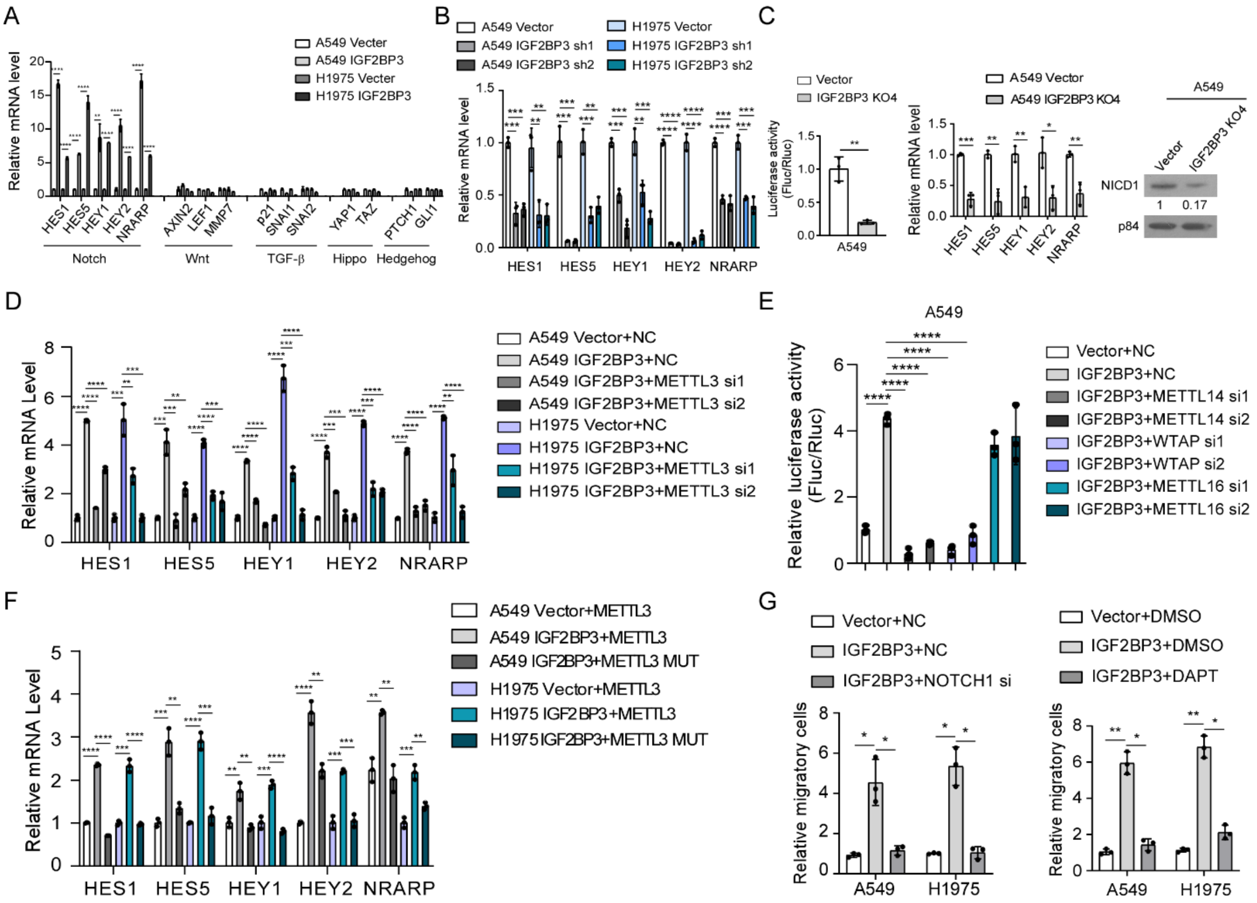

(A) Relative mRNA levels of downstream target genes along several metastasis-related signaling pathways in IGF2BP3-overexpressing and control LUAD cells. **(B)** Relative mRNA levels of downstream target genes of the Notch signaling pathway in IGF2BP3-silenced LUAD cells. **(C)** Luciferase assay, qRT-PCR assay and WB analyses examine alterations of the Notch signaling pathway in IGF2BP3-knockout (KO) cells. **(D)** qRT-PCR analyses show inhibitory effects of depletion of METTL3 on the downstream genes of IGF2BP3-promoted Notch signaling pathway. **(E)** Luciferase assay shows inhibitory effects of silencing several m^{6}A writers on the IGF2BP3-transactivated Notch signaling. **(F)** qRT-PCR analyses show catalytic mutation of METTL3 on the downstream genes of IGF2BP3-promoted Notch signaling pathway. **(G)** Indicated cells were plated in chambers with matrigel and assessed to show inhibitory effects of Notch1 silencing and γ-secretase complex inhibitor DAPT treatments on IGF2BP3-promoted metastasis-related traits. Invasive cells were quantified in five random fields. All experiments were repeated three times with similar results, and immunoblots in **(C)** are representative of three

independent experiments. Error bars represent the means ± SD derived from three independent experiments. Statistical analyses were performed using a two-tailed Student's t-test, *: P < 0.05, **: *P* < 0.01, ***: *P* < 0.001, ****: *P* < 0.0001.

Supplementary figure 3.

Supplementary figure 3

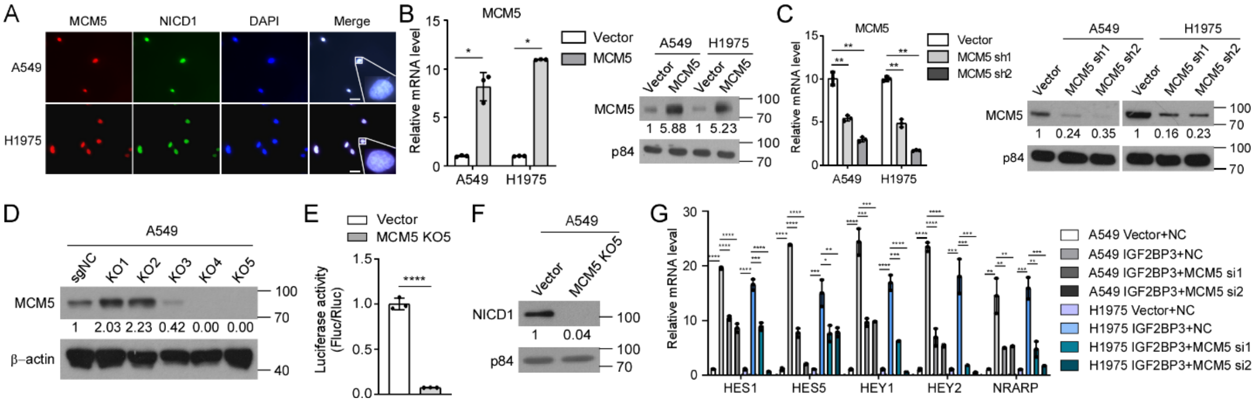

(A) Representative images of three independent experiments showing co-localization of MCM5 and NICD1 in LUAD cancer cell lines (insets: high-magnification images). Scale bar: 10 μ m. **(B)**-**(D)** Expression efficiencies of LUAD cell lines with stable MCM5 overexpressed **(B)**, knocked down **(C)** and knocked out **(D)**. **(E)** and **(F)** Luciferase assay **(E)** and WB analyses **(F)** examine alterations in the activity of Notch signaling in MCM5-knockout cells. **(G)** qRT-PCR assay shows inhibitory effects of MCM5 depletion on IGF2BP3-promoted downstream gene expression of the Notch signaling pathway. All experiments were repeated three times with similar results, and images/immunoblots in **(A)**-**(D)** and **(F)** are representative of three independent experiments. Error bars represent the means ± SD derived from three independent experiments. Statistical analyses were performed using a two-tailed Student's t-test, *: $P < 0.05$, **: $P < 0.01$, ***: $P < 0.001$, ****: $P < 0.0001$.

Supplementary figure 4.

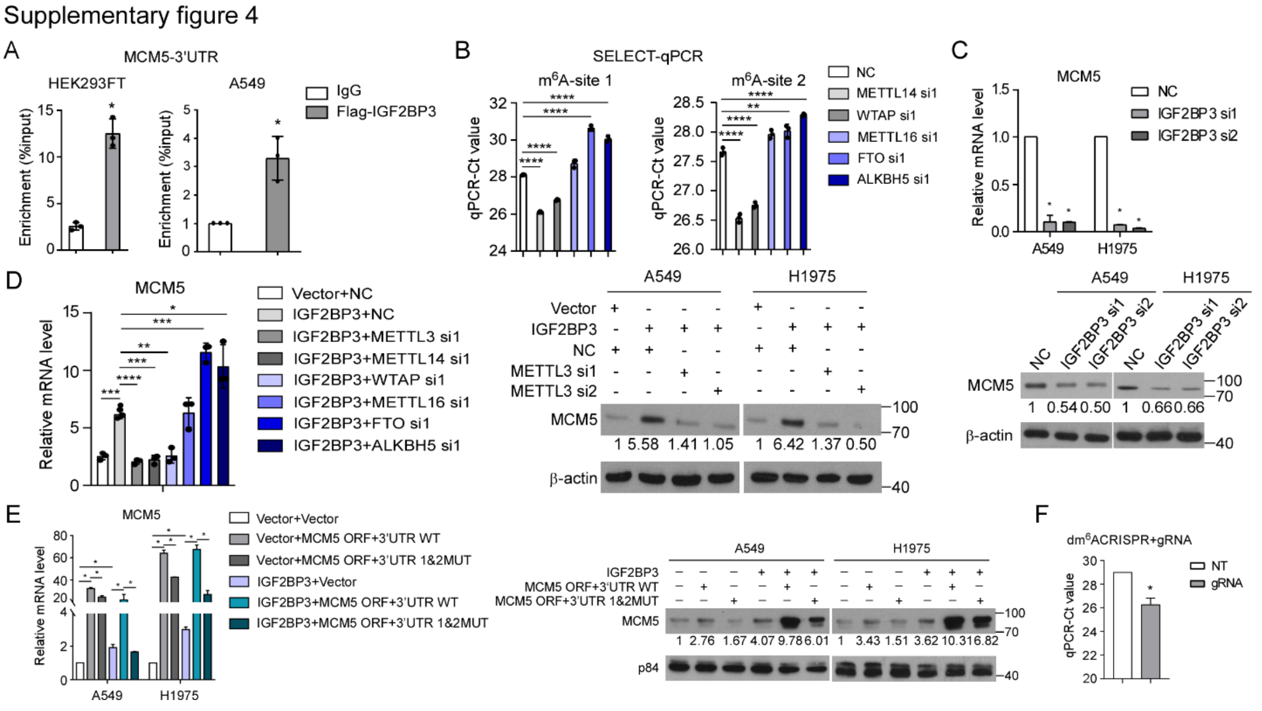

(A) RIP-qPCR detecting the binding ability of IGF2BP3 to MCM5 3'UTR in HEK293FT or A549 cells. **(B)** The threshold cycle (Ct) of qPCR shows SELECT results for detecting the m^{6}A site in mRNA 3'UTR of MCM5 in A549 cells with or without knockdown of m^{6}A regulators. **(C)** Determination of MCM5 mRNA (top) and protein (bottom) levels after IGF2BP3 depletions in LUAD cell lines by qRT-PCR and WB analyses. **(D)** Effects of silencing m^{6}A modifiers on MCM5 mRNA and protein expression in indicated LUAD cells. **(E)** MCM5 levels assessed after mutating MCM5 3'UTR in LUAD cell lines with or without stable expression of ectopic IGF2BP3 by qRT-PCR and WB analyses. **(F)** The threshold cycle (Ct) of qPCR shows SELECT results for detecting the m^{6}A site in 3'UTR of MCM5 in A549 cells transfected with dCas13b-ALKBH5 combined with gRNA control (NT) or gRNA. All experiments were repeated three times with similar results, and immunoblots in **(C)**, **(D)** and **(E)** are representative of three independent experiments. Error bars represent the means ± SD derived from three independent experiments. Statistical analyses were performed using a two-tailed Student's t-test, *: $P < 0.05$, **: $P < 0.01$, ***: $P < 0.001$, ****: $P < 0.0001$.

Supplementary figure 5.

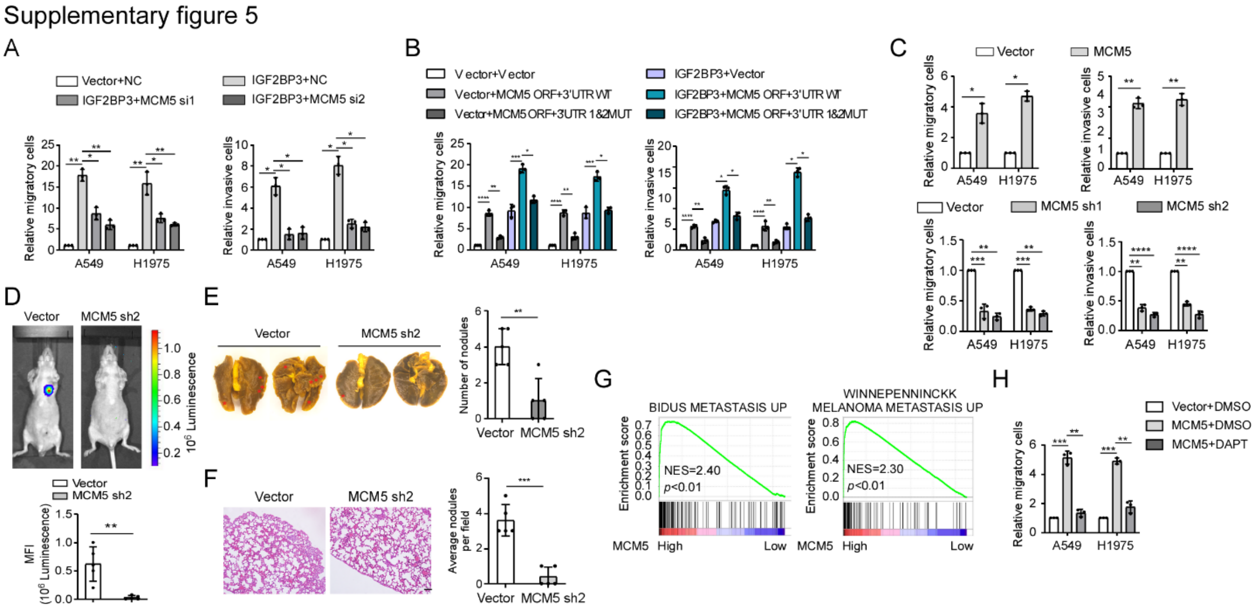

(A) and **(B)** Effects of MCM5 interference **(A)** or MCM5 3'UTR mutation **(B)** on average cell number of migration or invasion induced by over-expressing IGF2BP3. **(C)** MCM5-overexpressing or -silenced LUAD cells were plated in chambers to show metastasis-related traits by transwell assays. Migratory or invasive cells were quantified in five random fields. **(D)**-**(F)** Indicated A549 cells (1×10^6) were injected via tail vein. Representative bioluminescent images **(D)**, picric acid staining **(E)** and H&E staining **(F)** of lung metastases are shown. n=5. Scale bar: 50μm in **(F)**. **(G)** GSEA of the TCGA-LUAD datasets shows correlation between MCM5 and tumor metastasis-related signatures. **(H)** Average cell numbers of migratory cells with indicated treatments showing inhibitory effects of DAPT on MCM5-promoted metastasis-related traits. Migratory cells were quantified in five random fields. All experiments were repeated three times with similar results. Error bars represent the means ± SD derived from three independent experiments. Statistical analyses were performed using a two-tailed Student's t-test, *: $P < 0.05$, **: $P < 0.01$, ***: $P < 0.001$, ****: $P < 0.0001$.

Supplementary figure 6.

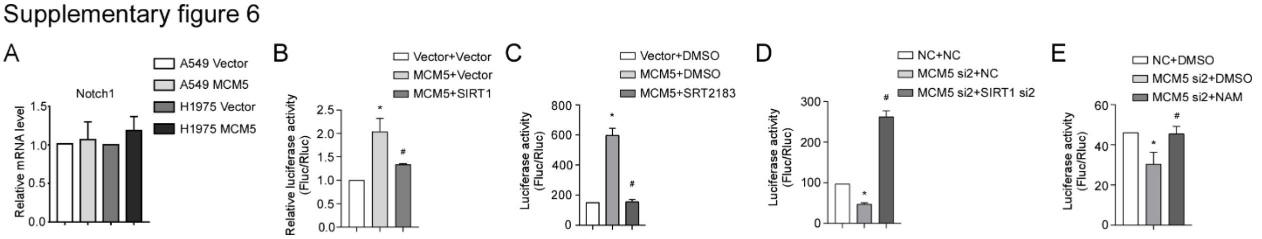

(A) qRT-PCR determined the expression of Notch1 mRNA in LUAD cell lines. **(B)** and **(C)** Using luciferase activity assay to examine the effects of SIRT1 over-expression **(B)** or treatments with SITR1 agonist SRT2183 **(C)** on Notch signaling activation cells with ectopic expression of MCM5. **(D)** and **(E)** Luciferase activity assay examines the effects of SIRT1 depletion **(D)** or treatments with NAM **(E)** on Notch signaling activation in MCM5-silenced cells. All experiments were repeated three times with similar results. Error bars represent the means ± SD derived from three independent experiments. Statistical analyses were performed using a two-tailed Student's t-test, *: $P < 0.05$, **: $P < 0.01$, ***: $P < 0.001$, ****: $P < 0.0001$.

Supplementary figure 7.

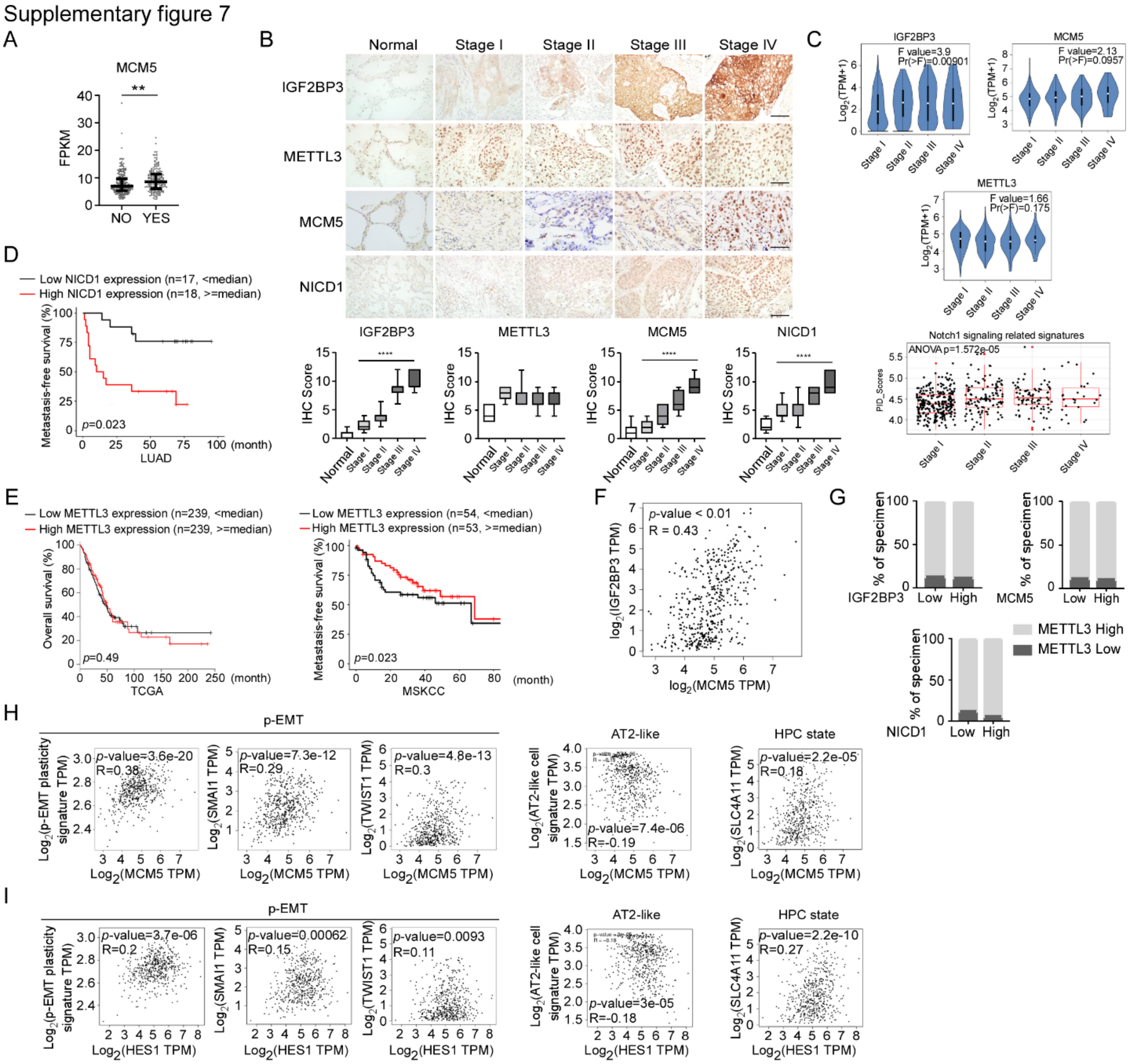

(A) Expression of MCM5 in 219 non-metastatic (NO) versus 185 metastatic (YES) samples from the TCGA cohort. Error bars represent the mean ± SD. Statistical analyses were performed using a two-tailed Student's t-test, **: P < 0.01. **(B)** Representative IHC images showing IGF2BP3, METTL3, MCM5 and NICD1 levels in normal lung or LUAD tumors from 14 patients with different clincal stages. Scale bar: 50µm. Statistical analyses were performed using One-Way ANOVA, ****: $P < 0.0001$. **(C)** Expression of IGF2BP3, METTL3, MCM5 and Notch signaling pathway grouped by clinical stages as analyzed from the TCGA LUAD datasets. Analyses were performed by R or the Gene Expression Profiling Interactive Analysis (GEPIA2, available at http://gepia2.cancer-pku.cn/). **(D)** Overall survival (OS) analysis of 35

LUAD patients according to median NICD1 expression. **(E)** Overall and metastasis-free survival analyses based on the TCGA LUAD and MSKCC datasets, respectively. **(F)** Correlation analysis of IGF2BP3 and MCM5 based on the TCGA LUAD datasets using the Gene Expression Profiling Interactive Analysis (GEPIA). **(G)** The expression association of METTL3, IGF2BP3, MCM5 and NICD1 according to their IHC intensities in consecutive slices from 14 cases of LUAD tissues. Statistical analyses were performed using chi-squared test. **(H)** and **(I)** Correlation analysis of MCM5 **(H)** or HES1 **(I)** with p-EMT plasticity, AT2-like and HPC state signatures in TCGA-LUAD cohort using GEPIA2.

Supplementary Table 1.

Clinicopathologic characteristics of patients enrolled for sample examination in 99 LUAD

		No of cases (%)
Gender	male	70 (70.7)
	female	29 (29.3)
Age (y)	＜60	120 (59.6)
	≥60	88 (40.4)
Clinical staging	I	30 (30.3)
	II	20 (20.2)
	III	41 (41.4)
	IV	8 (8.1)
T classification	T1	14 (14.1)
	T2	49 (49.5)
	T3	30 (30.3)
	T4	6 (6.1)
Lymphnode metastasis	No	41 (41.4)
	Yes	58 (58.6)
Distant metastasis	No	91(91.9)
	Yes	8 (8.1)

Supplementary Table 2.

Correlation between the clinical pathologic features of 99 LUAD and MCM5 levels

Characteristics		MCM5		P-value
		Low	High	
Gender	male	32	38	0.816
	female	14	15	
Age (y)	≤ 60	26	33	0.561
	＞60	20	20	
Clinical staging	I	20	10	0.02 *
	II	7	13	
	III	18	23	
	IV	1	7	
T classification	T1	8	6	0.111
	T2	23	26	
	T3	15	15	
	T4	0	6	
Lymphnode metastasis	No	23	18	0.106
	Yes	23	35	
Distant metastasis	No	45	46	0.045 *
	Yes	1	7	

Supplementary Table 3.

Correlation between the clinical pathologic features of 515 LUAD and MCM5 levels (Data from TCGA)

Characteristics		MCM5		P-value
		Low	High	
Gender	Male	116	123	0.564
	Female	141	135	
Age (y)	≤ 60	74	85	0.290
	＞60	174	163	
Clinical staging	I	154	122	0.007 **
	II	49	72	
	III	40	44	
	IV	8	18	
T classification	T1	91	78	0.640
	T2	132	145	
	T3	23	24	
	T4	10	9	
Lymphnode metastasis	No	180	152	0.003 **
	Yes	60	102	
Distant metastasis	No	179	167	0.022 *
	Yes	7	18	

Supplementary Table 4.

Univariate and multivariate analyses of various prognostic variables for overall survival in 515 LUAD from TCGA

	Univariate analysis		Multivariate analysis	
	P-value	Hazard ratio (95% CI)	P-value	Hazard ratio (95% CI)
Age (y)<br>≤60<br>＞60	0.274	1.195 (0.868-1.646)		
Gender<br>Male<br>Female	0.728	0.950 (0.713-1.267)		
Clinical staging<br>I<br>II<br>III<br>IV	＜0.001 **	1.659 (1.447-1.903)		
T classification<br>T1<br>T2<br>T3<br>T4	＜0.001 **	1.546 (1.288-1.855)	0.012 *	1.322 (1.062-1.645)
Lymphnode metastasis<br>NO<br>YES	＜0.001 **	2.548 (1.903-3.413)	0.025 *	1.638 (1.064-2.521)

Distant metastasis<br>NO<br>YES	0.009 **	2.044 (1.195-3.498)		
MCM5<br>Low<br>High	＜0.001 **	1.836 (1.366-2.468)	0.028 *	1.488 (1.044-2.122)
